# Systematic Phenotyping of a Large-Scale *Candida glabrata* Deletion Collection Reveals Novel Antifungal Tolerance Genes

**DOI:** 10.1371/journal.ppat.1004211

**Published:** 2014-06-19

**Authors:** Tobias Schwarzmüller, Biao Ma, Ekkehard Hiller, Fabian Istel, Michael Tscherner, Sascha Brunke, Lauren Ames, Arnaud Firon, Brian Green, Vitor Cabral, Marina Marcet-Houben, Ilse D. Jacobsen, Jessica Quintin, Katja Seider, Ingrid Frohner, Walter Glaser, Helmut Jungwirth, Sophie Bachellier-Bassi, Murielle Chauvel, Ute Zeidler, Dominique Ferrandon, Toni Gabaldón, Bernhard Hube, Christophe d'Enfert, Steffen Rupp, Brendan Cormack, Ken Haynes, Karl Kuchler

**Affiliations:** 1 Medical University Vienna, Max F. Perutz Laboratories, Department of Medical Biochemistry, Vienna, Austria; 2 Department of Microbiology, Imperial College London, London, United Kingdom; 3 Department of Molecular Biology and Genetics, Johns Hopkins University School of Medicine, Baltimore, Maryland, United States of America; 4 Molekulare Biotechnologie MBT Fraunhofer Institut für Grenzflächen- und Bioverfahrenstechnik IGB Fraunhofer, Stuttgart, Germany; 5 Department Microbial Pathogenicity Mechanisms, Hans-Knoell-Institute, Jena, Germany; 6 Friedrich Schiller University, Jena, Germany; 7 Center for Sepsis Control and Care, CSCC, Jena University Hospital, Jena, Germany; 8 Biosciences, College of Life & Environmental Sciences, University of Exeter, Exeter, United Kingdom; 9 Institut Pasteur, Unité Biologie et Pathogénicité Fongiques, Département Génomes et Génétique, Paris, France; 10 INRA, USC2019, Paris, France; 11 Université Paris Diderot, Sorbonne Paris Cité, Cellule Pasteur, Paris, France; 12 Bioinformatics and Genomics Programme, Centre for Genomic Regulation (CRG), Barcelona, Spain; 13 UPR 9022 du CNRS, Université de Strasbourg, Equipe Fondation Recherche Médicale, Institut de Biologie Moléculaire et Cellulaire, Strasbourg, France; 14 Institut für Molekulare Biowissenschaften, Universität Graz, Graz, Austria; 15 Universitat Pompeu Fabra (UPF), Barcelona, Spain; University of Rochester, United States of America

## Abstract

The opportunistic fungal pathogen *Candida glabrata* is a frequent cause of candidiasis, causing infections ranging from superficial to life-threatening disseminated disease. The inherent tolerance of *C. glabrata* to azole drugs makes this pathogen a serious clinical threat. To identify novel genes implicated in antifungal drug tolerance, we have constructed a large-scale *C. glabrata* deletion library consisting of 619 unique, individually bar-coded mutant strains, each lacking one specific gene, all together representing almost 12% of the genome. Functional analysis of this library in a series of phenotypic and fitness assays identified numerous genes required for growth of *C. glabrata* under normal or specific stress conditions, as well as a number of novel genes involved in tolerance to clinically important antifungal drugs such as azoles and echinocandins. We identified 38 deletion strains displaying strongly increased susceptibility to caspofungin, 28 of which encoding proteins that have not previously been linked to echinocandin tolerance. Our results demonstrate the potential of the *C. glabrata* mutant collection as a valuable resource in functional genomics studies of this important fungal pathogen of humans, and to facilitate the identification of putative novel antifungal drug target and virulence genes.

## Author Summary

Clinical infections by the yeast-like pathogen *Candida glabrata* have been ever-increasing over the past years. Importantly, *C. glabrata* is one of the most prevalent causes of drug-refractory fungal infections in humans. We have generated a novel large-scale collection encompassing 619 bar-coded *C. glabrata* mutants, each lacking a single gene. Extensive profiling of phenotypes reveals a number of novel genes implicated in tolerance to antifungal drugs that interfere with proper cell wall function, as well as genes affecting fitness of *C. glabrata* both during normal growth and under environmental stress. This fungal deletion collection will be a valuable resource for the community to study mechanisms of virulence and antifungal drug tolerance in *C. glabrata*, which is particularly relevant in view of the increasing prevalence of infections caused by this important human fungal pathogen.

## Introduction

*Candida glabrata*, a small, asexual, haploid yeast, is the second most frequent cause of candidiasis after *Candida albicans*, accounting for approximately 15%–25% of clinical cases [1]–[4]. *C. glabrata* forms part of the normal microbial flora in humans, but can cause serious infections in immunocompromised and hospitalized patients; antibiotic exposure and presence of central venous catheter devices, being additional important risk factors for disease development [2]. In contrast to the pleomorphic diploid *C. albicans* [5], *C. glabrata* is found clinically, exclusively as monomorphic yeast cells. It also lacks several attributes considered key mediators of fungal pathogenicity in other *Candida* spp, such as secretion of proteases and lipases [6], [7]. Despite the apparent absence of these well-known fungal virulence traits, *C. glabrata* remains highly pathogenic to humans. Hence, *C. glabrata* may rely upon distinct strategies and other virulence attributes to initiate infection, as well as to persist in infected patients.

Some traits that have been linked to the clinical importance and virulence of *C. glabrata* include an inherently elevated tolerance to azole antifungals [8]–[11]; the presence of a large repertoire of telomere-associated adhesins [12]–[18]; melanin-like pigment production [19]; adaptation to the acidic phagosomal environment and intra-phagosomal survival [20]–[22]; and Ace2-dependent components of the cell wall [23]. However, it is clear that the molecular basis of *C. glabrata* virulence is far from being completely understood.

*C. glabrata* clinical isolates generally exhibit a high inherent tolerance level to azole drugs [9]. While this trait has been extensively studied, the underlying mechanisms remain incompletely explained. Azole resistance can be acquired through increased expression of genes encoding ABC transporters (Cdr1, Pdh1, Snq2) or changes in their transcriptional regulatory system (Pdr1, Gal11) [24]–[28]. Mitochondrial dysfunction [29] and serum utilization via the putative sterol transporter Aus1 [30], [31] also impact the ability of *C. glabrata* to tolerate high azole levels. Notably, calcineurin signaling has been implicated in azole tolerance in *C. glabrata* [32]. It remains unclear if these are the only mechanisms driving azole resistance in *C. glabrata*. However, the clinical implications of this resistance demand alternative antifungals for effective treatment of *C. glabrata* infections in patients, especially since *C. glabrata* infections are globally rising, sometimes accounting for more than 30% of clinical cases [33].

Echinocandins such as caspofungin (CF), anidulafungin and micafungin are a relatively new class of effective yet high-cost antifungal drugs targeting fungal 1,3-β-D-glucan synthases and thereby impairing cell wall integrity [34]. Mutations in subunits of glucan synthases can render fungi resistant to echinocandin [34]–[37]. Surprisingly, even ectopic overexpression of the *C. albicans* Cdr2 efflux ABC transporter gene in both laboratory strains and clinical isolates markedly increases CF tolerance [38]. Furthermore, the Hsp90 heat shock protein has also been identified as a regulator of echinocandin tolerance acting through calcineurin signaling [36], [39]. However, a better understanding of molecular mechanisms modulating echinocandin susceptibility is necessary, since it may facilitate targeted drug discovery, especially in the case of emerging resistant strains [40]. Importantly, an increase in the number of echinocandin resistant *C. glabrata* clinical isolates has been reported recently, implying that many genes can contribute to echinocandin tolerance [41].

Reverse genetics coupled with global functional profiling has proven a powerful approach to identify genes required for specific phenotypes. Functional genomics studies in the non-pathogenic yeast *S. cerevisiae* have provided the starting point to decipher genotype-phenotype relations as they enabled answers about fundamentally important questions concerning complex genetic interactions and the genetic landscape of yeast. These approaches also unraveled stress response mechanisms and provided new insights into drug susceptibility and morphogenesis [42]–[49]. Heroic efforts by a few groups have recently resulted in highly useful genome-scale deletion collections of the major pathogen *C. albicans* [50]–[52] and *Cryptococcus neoformans* [53], enabling the identification of novel virulence genes and further demonstrating the power of a functional genomics approach.

Here, we have adapted a semi-automated approach [51] for constructing gene deletions to generate a collection of individually bar-coded strains in the sequenced *C. glabrata* strain ATCC2001 [54], each lacking one defined open reading frame. We took advantage of this library to undertake the first systematic functional-genomic and phenotypic analysis of *C. glabrata*, in particular examining the response to traits putatively implicated in virulence and antifungal tolerance of this human pathogen of increasing importance and prevalence. We performed a series of growth assays in distinct media to determine the impact of gene deletion on fitness. We then determined the susceptibility of the collection to major antifungal compounds (including azoles and caspofungin) and various other cell wall-damaging compounds. Finally, we investigated the effect on cellular morphology on fitness *in vitro*, and the ability to form biofilms. This enabled us to generate the first large-scale chemogenetic and phenotypic profile of *C. glabrata*. Our analysis revealed numerous novel genes implicated in stress response, cell wall homeostasis, growth morphology and fitness. Most importantly, we discovered numerous novel genes implicated in susceptibility to echinocandins, demonstrating the usefulness of this deletion collection for the functional analysis of virulence-related, as well as clinically relevant traits, including the discovery of novel antifungal target genes.

## Results

### Gene selection

Using a large-scale phylogenetic approach across many fungal species [55], we identified 1047 putatively non-essential candidate genes in *C. glabrata* representing functional GO categories such as environmental stress sensing and signaling (MAPK pathways, TOR, RIM, PKA), transcriptional regulation, antifungal drug resistance (PDR network, membrane permeases), cell wall structure and homeostasis (glucan, mannan, chitin synthesis, glycosylation, adhesins, glycosylphosphatidylinositol (GPI)-anchor), chromatin and histone modification, iron metabolism and metal sensitivity, as well as peroxisome biogenesis. We also selected genes lacking obvious orthologues in *S. cerevisiae* (Table S1).

### Parental recipient strains

To enable the rescue of deletion phenotypes, and to facilitate double- or triple mutant construction, we engineered a triple-auxotrophic recipient strain in the sequenced strain *C. glabrata* ATCC 2001 [54]. We used the dominant recyclable nourseothricin resistance marker *SAT1* [56] to replace the coding sequences of *HIS3*, *LEU2* and *TRP1* (Figure S1A). The repeated use of this marker cassette resulted in the new *C. glabrata* background recipient strain for deletions referred to as HTL (*his3*Δ::FRT *leu2*Δ::FRT *trp1*Δ::FRT), as well as all possible isogenic single deletions and all combinations of double deletion strains (Table S2). We also constructed a bar-coded version of the HTL strain, *C. glabrata* HTL reference, by inserting 20 bp barcodes flanking the *trp1* locus (Table S2).The transcription factor (TF) mutants were made in a *his3* derivative of ATCC2001, the majority using a codon-optimized version of the *NAT1* marker [57]. We avoided the use of the *URA3* marker in *C. glabrata*, since it is known to alter virulence properties of *C. albicans* [58], [59]. The HTL strain displayed similar growth properties and rates as the parental strain, on both minimal and full media (Figure S1C). Importantly, the auxotrophic markers did not significantly influence growth *in vitro*, or the survival in immunocompetent mice when compared to the parental wild type strain [60]. While the growth behavior of *C. glabrata* HTL was largely unchanged, these cells reached a slightly lower maximal cell density when growing in minimal media when compared to wild type cells (Figure S1C).

### Gene deletion strategy

**Figure 1:**
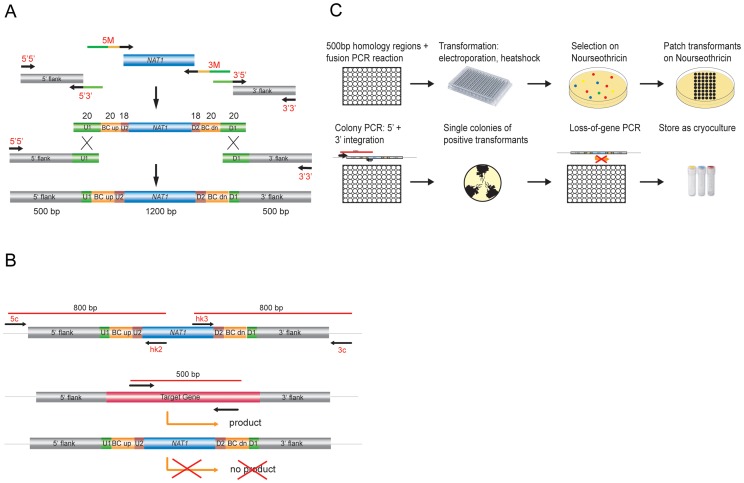
Generation of *C. glabrata* mutants and systematic phenotypic analysis. (a) Generation of gene deletion constructs by fusion PCR using the dominant selectable marker *NAT1*. A set of two times 96 unique barcode sequences was integrated in oligonucleotides to amplify the marker fragment and to add overlap sequences. (b) Transformants were verified by colony PCR for correct integration on the 5′ and 3′ junction and checked for absence of the target ORF. (c) Overview of the construction of the gene deletion strain library.

Targeted gene disruption with short-homology flanking regions, as was done to construct the *S. cerevisiae* knock-out library, is inefficient in *C. glabrata*. Higher targeting efficiency requires the use of ≥500 base pair (bp) flanking regions [61]. Therefore, to maximize efficiency of gene replacement, we adapted and automated the fusion PCR technique [51] to generate gene deletion constructs containing ∼500 bp homologous flanking regions for every gene, fused to the dominant marker *NAT1* and flanked by unique barcode identifiers (Figure 1A). We employed a limited set of barcode sequences selected from those successfully used in the *S. cerevisiae* genome deletion project [43], [49]. These barcodes enable quantification and tracking of single mutants in pool experiments. A complete list of all barcode sequences corresponding to each deleted ORF in the collection is given in Table S3.

*C. glabrata* recipient strains were transformed with these deletion cassettes using a modified 96-well format electroporation protocol. All resulting nourseothricin-resistant transformants were tested for correct genomic integration by colony PCR to verify both 5′ and 3′ junctions (Figure 1B). Single colonies of up to six verified transformants for each gene were isolated; the absence of the corresponding gene from the genome was confirmed using another PCR-based step and confirmed gene deletion strains were cryo-preserved (Figure 1B, C). A total of 24 PCR-verified gene deletion strains were randomly selected and subjected to Southern blot analysis to confirm correct genomic replacements (data not shown).

In all, we successfully deleted 619 of the 1047 genes initially selected for inactivation, yielding a total of 1601 independent unique deletion strains (Table S3; http://funpath.cdl.univie.ac.at), representing 59% of the selected genes and about 12% of the entire *C. glabrata* genome. Notably, for 77 genes only one deletion was obtained, while the majority of deletions result from two (246) or three (224) independent transformation and thus genomic removal events (Table S3). We assume that a large fraction of the genes that we failed to inactivate is a consequence of inefficient homologous targeting, rather than a true representation of the frequency of essential genes.

### Validation of screening conditions

Fungal pathogens need to adapt to diverse host immune defense mechanisms and environmental stresses. Relevant stress conditions employed include perturbations of cell wall integrity, osmostress during phagolysosome maturation and growth at elevated temperature as well as often exposure to antifungal drugs. An efficient adaptation to particular stress conditions may also include formation of biofilms. We therefore phenotypically profiled the mutant collection to identify genes implicated in the response to host-mimicking adverse conditions. First, we carried out a preliminary pilot screen using a small set of selected deletion mutants displaying known phenotypes. Mutants lacking genes of the high osmolarity glycerol (HOG) pathway, the cell integrity protein kinase C (PKC) pathway and the pleiotropic drug resistance (PDR) network were tested for growth under conditions known to affect the corresponding mutants in *S. cerevisiae*. As expected, lack of *PBS2*, encoding the central MAPK (mitogen-activated protein kinase) of the HOG pathway [62], resulted in severe osmosensitivity (Figure S2A). Likewise, azole hypersensitivity was observed in *cdr1* and *pdr1* strains (Figure S2B) [24]–[26], [28], [63], [64]. Finally, as previously shown cells lacking the Slt2 kinase of the PKC pathway [65], [66] displayed drastic hypersensitivities to CF (Figure S2C).

Following these initial experiments, which served to establish and validate screening parameters, the collection of 1601 gene deletion mutants was subjected to extensive phenotypic profiling in four independent laboratories using the same conditions. The deletion library was screened for various phenotypes, including growth defects in YPD at 30°C, defects in biofilm formation, sensitivities against antifungal drugs (azoles, amphotericin B (AmB)) in liquid medium, and cell wall-perturbing agents (Congo Red (CR), Calcofluor White (CW) and CF), as well as heat stress (42°C) and osmostress (NaCl) on solid media. Furthermore, colonies were also inspected for obvious morphology alterations. A total number of 196 mutants showed phenotypes different from the wild type control for at least one condition tested. A summary of all of these mutants with their phenotypes is provided in Table S4.

### Growth and fitness defects

**Figure 2:**
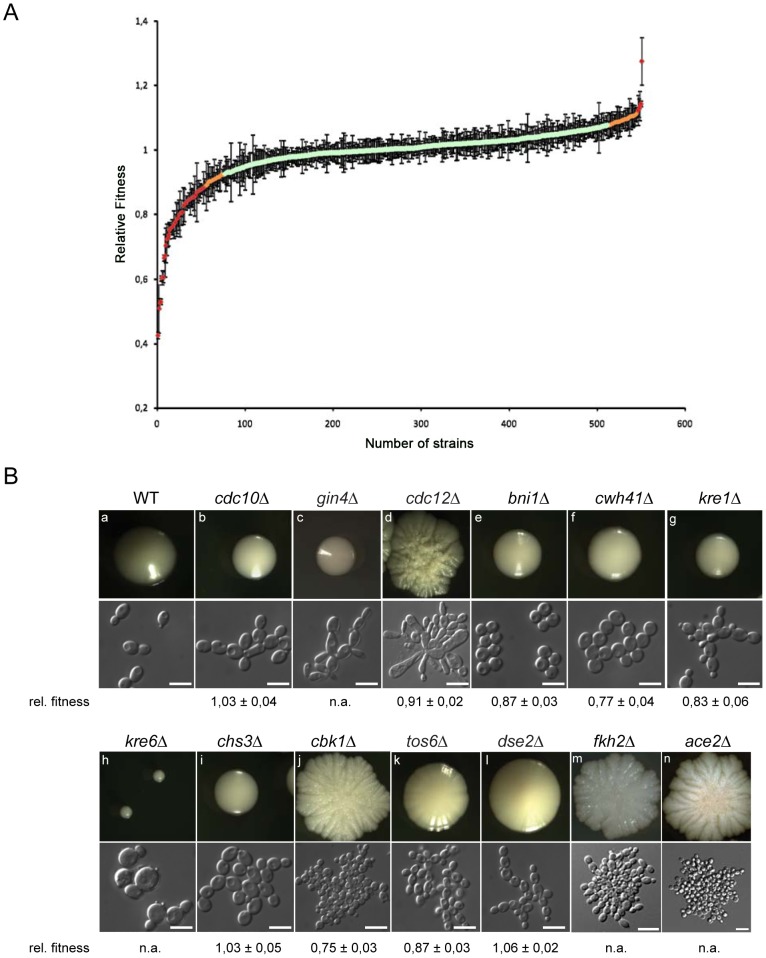
Relative fitness distribution and morphology of *C. glabrata* gene deletion strains. (a) Wild type and mutant strains were grown in rich medium at 30°C and doubling times were recorded. The median doubling time of the wild type *C. glabrata* ATCC2001 strain under these conditions was 63.9 min while the median for mutant strains was 68.1 min. For each strain, the relative fitness was calculated using doubling times from at least two independent cultures of 1–6 independent mutants. Strains that showed strong variations in relative fitness between independent mutants or independent cultures were omitted from further analysis (Table S5). Data were obtained for 503 knock-out mutants (Table S6). (b) Colony and cell morphologies of *C. glabrata* deletion strains. Different types of distinct cell and colony morphologies were found. Cell morphology classes: ellipsoid (a), chains (b, f, g, i, k, l), elongated (c, d), large clumps (d, j, m, n), round (e), large and round (h); colony morphologies: smooth (a, b, c, e, f, g, i), small (h), slightly wrinkled (k, l), wrinkled (d, j, m, n). WT = HTL background strain; white bars correspond to 10 µm.

Changes in the pathogenic potential of a fungus may be associated with a gain or loss of *in vitro* or *in vivo* growth, influencing the ability to efficiently replicate in the host or to withstand antifungal treatment [67]. Thus, we measured the growth rates of individual mutants in the deletion collection in YPD at 30°C. For each deletion strain, a relative fitness index [68] was calculated using the doubling times from at least two independent cultures of 1–6 independent mutants, and compared to the doubling time of all other strains. Notably, we identified 57 deletion mutants showing strong variations in relative fitness between independent cultures (Table S5), all of which were omitted from further analysis along with deletions that were present in duplicate. Hence, reproducible fitness data could be obtained for 503 unique mutants (representing 1125 deletions). Out of these gene knock-outs, 402 had a relative fitness index within two standard deviations (SD) of the average relative fitness (Figure 2A, Table S6). These data suggested that the corresponding genes are not necessary for efficient planktonic growth of *C. glabrata* in YPD at 30°C. However, 70 strains showed a significant fitness defect (doubling time of ≥2 SD below the average fitness). Interestingly, another 34 deletions showed a gain in fitness (doubling time of ≥2 SD above the average fitness) under these growth conditions (Figure 2A, Table S7).

A total of 13 deletion strains showed obvious alterations of both colony and cellular morphology (Figure 2B). Five of these mutants were also shown to exhibit fitness defects *in vitro* (Table S6). Most morphology mutants grew as small or large wrinkled or smooth colonies. Microscopic inspection of deletion mutant phenotypes allowed for further classification into different cell morphology classes, including round cells (*dse2*, *tos6*, *ace2*, *chs3*, *bni4*, *cwh41*), giant cells with obvious structural defects (*cdc21, cbk1, kre6*), cells with pseudohyphal-like elongated morphologies (*gin4*), and as pearl-string-like cells connected to each other as for *dse2* mutants (Figure 2B). Notably, except for pseudohyphal morphologies [69], [70] or small round cells as for *ace2* [23], many of these morphological alterations have not been described in *C. glabrata* to date.

### Biofilm formation

**Figure 3:**
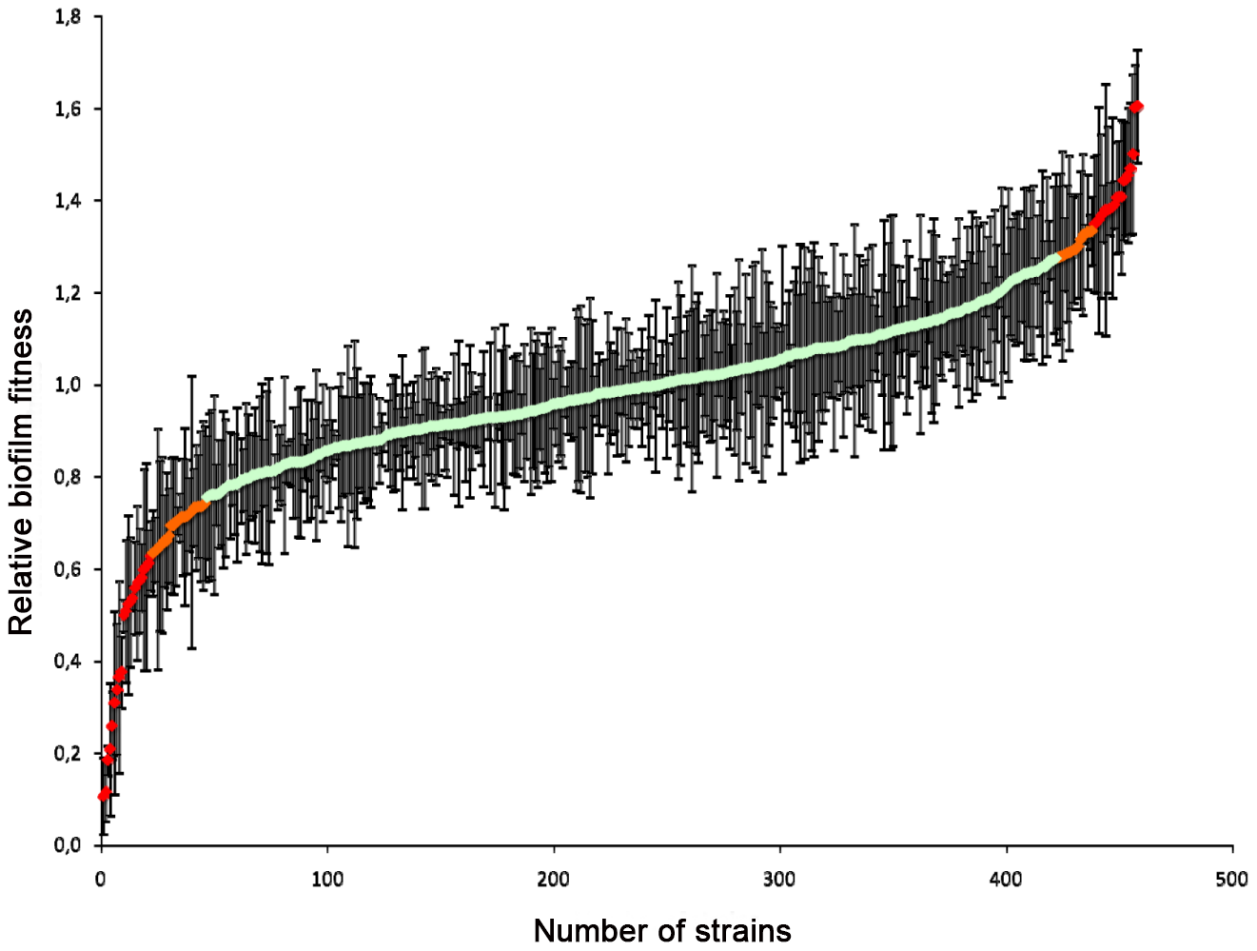
Relative biofilm fitness distribution. Wild type and mutant strains were induced to form biofilms in 96-well polystyrene plates in minimal medium at 37°C and the biofilm biomass was quantified using fluorescein diacetate (FDA). For each strain, a relative biofilm fitness was calculated based on FDA hydrolysis data from at least two independent cultures. Data were obtained for 420 knock-out mutants (Table S9). Strains that showed strong variations in relative fitness between independent mutants or independent cultures were omitted from further analysis (Table S8).

Biofilm formation on indwelling medical devices represents a significant risk for invasive infections by *Candida spp*, since biofilms display both increased drug tolerance and represent a persistent source of shedded cells that disseminate via the blood stream [71]–[73]. Hence, mutants in the deletion collection were scored for their ability to form biofilms. Strains were induced to form biofilms in 96-well polystyrene plates in minimal medium at 37°C; the biofilm biomass was quantified by determining the metabolic activity using a fluorescein diacetate (FDA) accumulation assay [74], [75]. For each strain, a relative biofilm-forming index was calculated using FDA hydrolysis data from at least two independent cultures of the independent deletion mutants for each gene (see Materials & Methods; Table S8, S9, S10). Independent deletions of the same gene including duplicates showing strong variations in relative biofilm formation between independent assays or independent cultures (Table S8) were omitted from further analysis, leaving 420 deletions for which biofilm production was analyzed in detail (Figure 3; Table S9).

Out of these, 341 gene deletions resulted in a relative biofilm forming index within two standard deviations (SD) of the average relative biofilm-forming capacity (Figure 3; Table S10), suggesting that the corresponding genes do not contribute to biofilm formation in minimal medium at 37°C. Isolates for the 46 gene deletions that resulted in an alteration in biofilm formation (i.e. a biofilm fitness score 2 SD below or above the average relative biofilm-forming index) but not of planktonic growth at 30°C were retested in quadruplicate for their ability to form biofilms. Moreover, the planktonic growth rate of these strains in minimal medium at 37°C was monitored to assess possible effects of the higher incubation temperature on biofilm formation. Notably, four of the corresponding deletion strains, namely those corresponding to *FKH2*, *PKH2*, *SNF1* and *ACE2*, also displayed temperature-sensitive phenotypes and may therefore not be solely biofilm-specific. Yet, this analysis identified 14 gene deletions that resulted in significant defects in biofilm formation but no significant defect in planktonic growth, including *AVO2, BCY1, CCW12, CCH1, CNB1, DCW1, GAS1, MHP1, PKH2, SLM1, SUB1, UTP14, YNL300W and YOR1* (Table S9). Remarkably, we identified 11 gene knock-outs resulting in a gain in biofilm formation without an increase in planktonic growth, including *BPH1, GAL11, GPB2, MIG1, PEX2, SSN2, SSN8, STE20, YAP6, YDR134C and YVC1* (Table S9).

### Susceptibility to azole and AmB antifungal drugs

Azoles and AmB remain the most common drugs for treating fungal infections. The inherently reduced azole susceptibilities of most *C. glabrata* clinical isolates is considered a major contributor to the increasing clinical prevalence of this pathogen [76]. While this is mainly the consequence of transcriptional upregulation of the *CDR1* and *PDH1* (*CDR2*) encoding membrane efflux pumps or gain-of-function mutations in the *PDR1* regulator [28], additional mechanisms may play a role. The polyene AmB is thought to impair membrane function by binding to ergosterol, resulting in cellular leakage of cytoplasm [77]. While *C. glabrata* can develop AmB tolerance [78], [79], the underlying molecular mechanisms remain obscure. We have thus used the *C. glabrata* deletion collection to identify genes modulating azole as well as AmB susceptibility.

**Figure 4:**
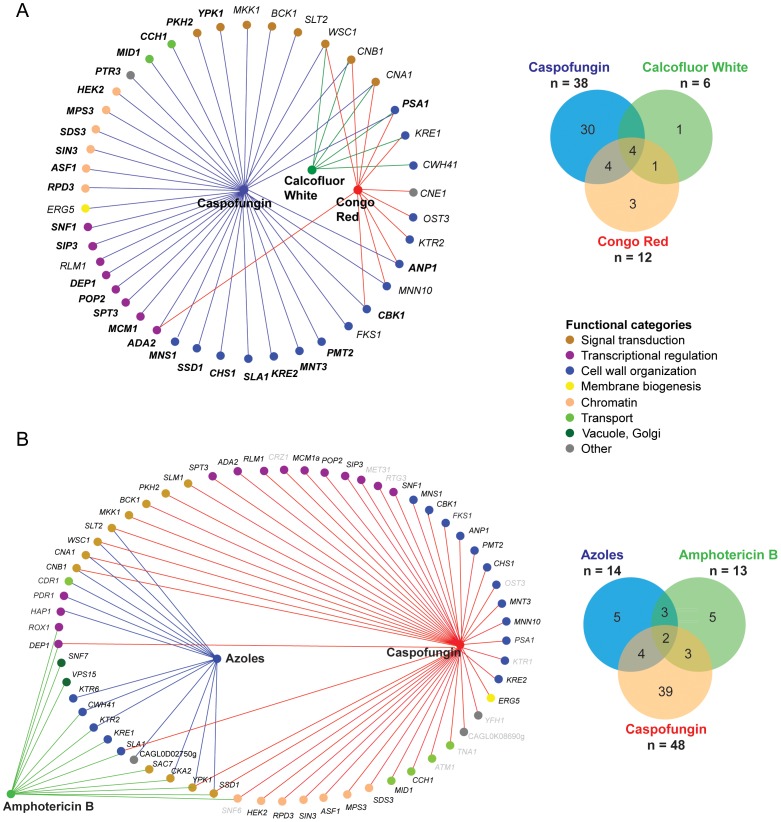
Classification of *C. glabrata* chemogenetic profiles. (A) Overlap between the chemical-genetic profile of CF (38 genes), CR (12 genes) and CW (6 genes). 28 genes (labeled in bold letters) were novel CF tolerance genes, since they have not been previously associated with echinocandin hypersensitivity in *S. cerevisiae* or *C. albicans*. (B) Overlap between the chemical-genetic profile of CF, azoles and AmB. 61 genes displayed an increased sensitivity to CF (38 genes plus 10 genes with weak sensitivity), azoles (14 genes; fluconazole and voriconazole) and AmB (13 genes). The mutant strain collection was screened in a 96 well microplate format using an endpoint assay and medium was supplemented with 5 µg/ml fluconazole, 100 ng/ml voriconazole and 1,5 µg/ml AmB. The OD_600_ was determined after 24 and 48 hours of incubation at 30°C. Grey-colored genes only display a weak sensitivity phenotype or were excluded from further analysis due to strong variations in the screening. Nodes represent compounds or genes and edges indicate chemical-genetic interactions. Gene nodes are color-coded according to GO annotation. Venn diagrams summarize distribution of genes affecting resistance to one of the three compounds.

A total of 14 deletion strains displayed marked hypersensitivities to azoles such as fluconazole and voriconazole albeit to a different extent (Figure S5A, C; Figure 4B). Moreover, 13 mutants were hypersusceptible to AmB (Figure S6A, Figure 4B). The corresponding mutants were retested using microdilution assays to quantify their IC_50_ values (Figure S5B, D; Figure S6B). The majority of the 14 azole-sensitive strains were sensitive to both fluconazole and voriconazole (Figure S5A, C), while 6 strains (*ktr2*, *cwh41*, *ssd1*, *ktr6*, *hap1* and *slt2*) appeared more sensitive to voriconazole. Notably, the strain lacking the *KTR2* gene encoding a mannosyltransferase [80] displayed the most significant voriconazole-specific hypersensitivity (Figure S5C, D). As expected, deletion of either the *PDR1* transcription factor or its target *CDR1* efflux pump resulted in marked azole hypersensitivity (Figure 4B, Figure S2, S8D). Furthermore, calcineurin pathway mutants such as *cna1* and *cnb1* also displayed pronounced azole hypersensitivities, as also shown by previous reports [32], [81]. Several additional signaling mutants (*slt2*, *wsc1*, *ypk1*, *cka2*), as well as cell wall mutants (*ktr2*, *cwh41*, *ssd1*, *ktr6*) displayed slight to intermediate azole hypersensitivities (Figure S5A, B; Figure 4B). Among the 13 AmB-sensitive strains, the five genes displaying the most pronounced susceptibilities play diverse roles in phospho- and sphingolipid signaling, including *YPK1*, *CKA2*, *DEP1*, *SNF6* and *VPS15*.

### Cell wall stress, osmostress and heat sensitivity

**Figure 5:**
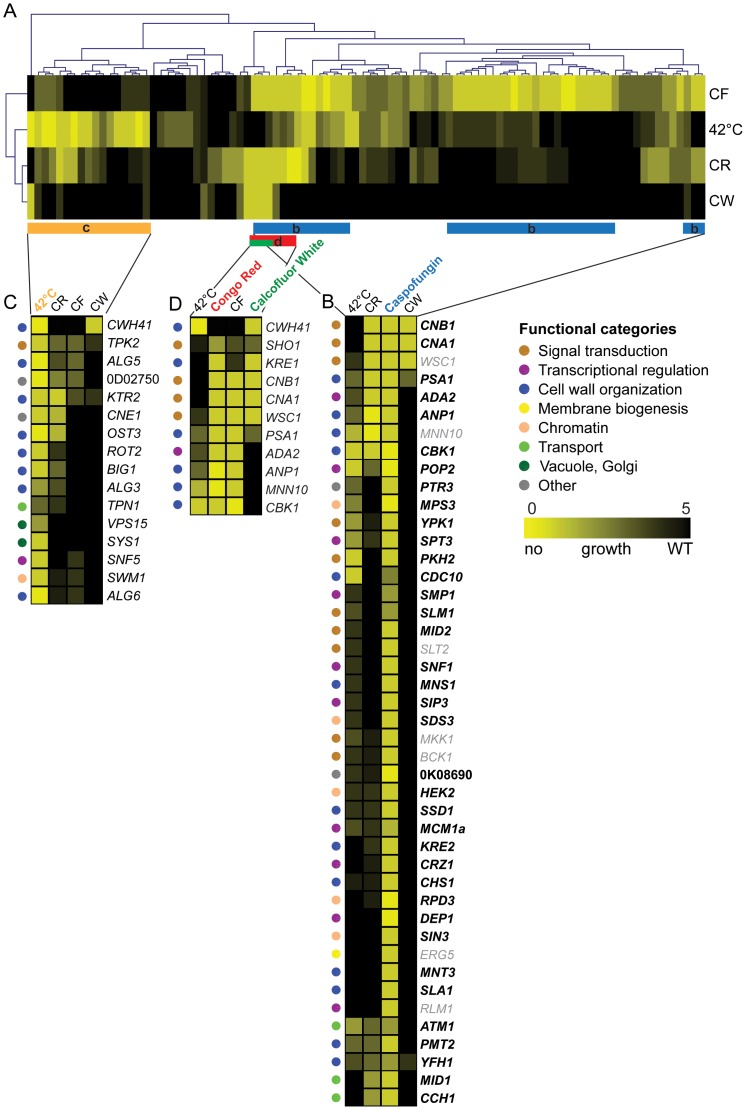
Clustering of susceptibility data of the *C. glabrata* gene deletion collection. The set of mutants was screened on plates for hypersensitivity to four distinct stress conditions (CF, CR, CW and 42°C heat stress) using serial dilution assays on agar plates. (A) Two-dimensional hierarchical cluster plot of chemical-genetic profiles. On the horizontal axis genes are listed and stress conditions on the vertical axis. Interactions are shown in yellow depending on the degree of growth sensitivity (yellow = no growth, black = WT growth). Stress conditions and genes are clustered by the similarity of their interactions. (B) The clusters of genes are enlarged to highlight the chemical-genetic interaction profile of Caspofungin (blue bar in ‘a’ labeled ‘b’). (C) A section of the cluster (orange bar labeled ‘C’) involved in heat stress (42°C) is enlarged. (D) Genes implicated in sensitivity to the cell wall-perturbing agents CR and CW (red and green bar labeled ‘D’).

The fungicidal echinocandins stand out as the most efficient clinically used drugs that block cell wall glucan biogenesis. Thus, we subjected the deletion collection to profiling for susceptibilities to the caspofungin (CF) echinocandin, as well as other cell wall stressors such as CR and CW (Figure 4, Figure 5, Figure S7). A total of 12 mutants were strongly hypersensitive to CR, and six to CW (Figure 4A; Figure S7C). Unsurprisingly, deletion of genes encoding functions implicated in cell wall integrity or polarity (*PSA1, KRE1, CWH41, OST3, KTR2, MNN10, ANP1, CBK1*) conferred hypersensitivity to CR and/or CW. Strikingly, we identified 48 mutants with altered CF susceptibilities (Figure 4A, B), 38 of which were strongly hypersensitive, while another 10 were mildly CF-sensitive, including *RTG3*, *YFH1*, *TNA1*, *ATM1*, *SNF6*, *CRZ1* (Figure 4, Figure S7).

**Figure 6:**
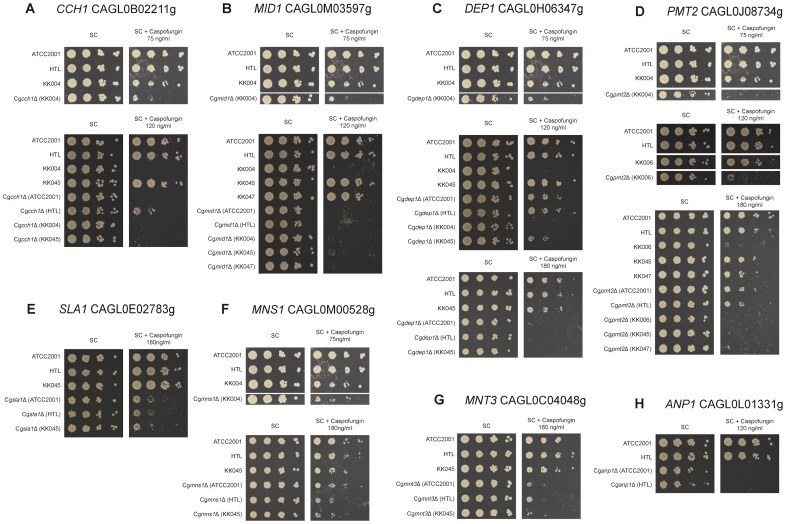
Caspofungin sensitivity of *C. glabrata* clinical isolate deletion strains. Sensitivities of deletion strains constructed in clinical isolates (KK004, KK006, KK045 and KK047) or in the ATCC2001 strain were tested for caspofungin (CF) susceptibility on plates. Strains were spotted in serial dilutions on synthetic agar medium supplemented with the indicated CF concentrations and growth was monitored for 3 days at 30°C. Screening was performed for (A) *CCH1*, (B) *MID1*, (C) *DEP1*, (D) *PMT2*, (E) *SLA1*, (F) *MNS1*, (G) *MNT3* and (H) *ANP1*.

To identify the functionally overlapping mutants, we used hierarchical clustering of stress-induced phenotypes, including heat, CF, CR and CW, identifying some 106 putative chemical-genetic interactions (Figure 5). The clustering approach identified three subsets of deletion mutants displaying distinct but partially overlapping hypersensitivities to high temperature, CR, CW and CF (Figure 5). Remarkably, the profiling analysis revealed some 28 novel CF tolerance genes, none of which had previously been associated with echinocandin hypersensitivities in *S. cerevisiae*, *C. albicans* or in other fungal pathogens (Figure 4, Figure 5). Importantly, CF sensitivity phenotypes of a subset of these mutants were confirmed in different strain backgrounds, including unrelated clinical isolates (Figure 6, Figure S8). Furthermore, restoration of the wild type phenotypes upon reintroduction of the corresponding genes confirmed that the observed caspofungin sensitivity in deletion strains is specifically caused by the lack of the respective gene (Figure S9). Notably, the group of genes affecting CF sensitivity contained several genes operating in the PKC cell integrity signaling pathway (*WSC1*, *SLT2*, *BCK1*, *MKK1*), in calcium/calcineurin signaling (*CNA1*, *CNB1*, *MID1*, *CCH1*), general cell wall homeostasis, including mannosylation and glycosylation (*MNN10*, *ANP1*, *MNS1*, *MNT3*, *PMT2*, *PSA1*, *KRE2*), as well as transcriptional regulators (*RLM1*, *DEP1*, *POP2*, *SPT3*, *MCM1*, *SIP3*, *SNF1*). Interestingly, deletion of several genes encoding components of the chromatin and histone modification machinery (*RPD3*, *HEK2*, *MPS3*, *SDS3*, *SIN3*, *ASF1*) also modulated CF susceptibility, suggesting an important regulatory role for chromatin in controlling surface homeostasis and CF susceptibility, as recently demonstrated for the *C. albicans* Hat1 acetyltransferase [82]. The removal of only four genes (*WSC1*, *CNB1*, *CNA1* and *PSA1*) resulted in sensitivity to all three cell wall stressors (Figure 4A), confirming the pivotal roles PKC and calcineurin signaling pathways play in sensing and maintaining cell wall homeostasis in fungal pathogens [36], [83], [84].

Notably, the heat stress profiling on plates identified several genes implicated in cell wall biogenesis and organization, including *CWH41*, *ALG5*, *OST3*, *ALG6*, *KTR2*, *BIG1*, *CBK1* and *ALG3*. Interestingly enough, this gene set showed some overlap with the CF, CR or CW gene clusters (Figure 5), suggesting that heat stress triggers partially overlapping signaling response pathways, ranging from stress and cell integrity signaling to cell wall homeostasis and membrane lipid perturbation.

Moreover, we also identified remarkably strong osmosensitivity phenotypes caused by the loss of genes that have never been linked to osmostress in other fungi before, including *ATM1*, *ARB1*, *KRE1*, *ANP1*, *MPS3* and *PTR3* (Figure S3). These data suggest that even the highly conserved fungal osmosensing pathway has acquired additional and/or novel components in *C. glabrata*.

## Discussion

*C. glabrata* is an important human fungal pathogen and, after *C. albicans*, the second-most frequent cause of candidiasis, causing 15–30% of infections in humans [1]–[4]. Although *C. glabrata* appears similar to *S. cerevisiae* concerning gene synteny and conservation it is an obligate haploid, and the lack of a sexual cycle and elevated rates of non-homologous recombination have prevented a systematic genetic analysis. Importantly, the decreased sensitivity of *C. glabrata* clinical isolates to azole antifungals is at least in part responsible for its clinical significance [33], and recent clinical reports suggest that azole antifungal resistance in *C. glabrata*, including multidrug resistance, is emerging at a rapid pace, thus posing an important challenge to therapeutic management [40], [85].

To aid in addressing these issues, as well as to better understand virulence properties, we have generated a large-scale deletion collection of *C. glabrata* representing almost 12% of the genome. This deletion mutant collection that comprises 619 unique strains each lacking a single gene has several advantages over known transposon-based mutant collections [86], since each ORF is completely removed. Thus, gene dosage effects or dominant phenotypes from truncated protein variants are excluded. Likewise, aberrantly expressed proteins due to insertion in promoters, which may result in partial loss or gain-of-function for the corresponding gene product, are precluded. The deletion collection was subjected to extensive phenotypic profiling, with a particular emphasis on susceptibility to major antifungal drugs, growth and morphology phenotypes, stress response pathways, cell wall biogenesis as well as biofilm formation, which has been associated with the initiation and development of candidiasis [87]. Importantly, we show here the feasibility to generate a genome-scale bar-coded gene deletion library in *C. glabrata*, revealing novel genes contributing to fitness, biofilm formation, antifungal drug resistance and several genes implicated in general cell wall homeostasis. We found 196 mutants sensitive to at least one of the tested conditions. In summary, 21% of the tested mutants display altered fitness phenotypes and 19% show alterations in biofilm formation, while only 2% exhibit abnormal cell and colony morphology. Approximately 16% of the mutants show severe stress-related phenotypes, including hypersensitivity to several antifungal drugs, heat and osmostress conditions.

### General fitness defects

*C. glabrata* and *S. cerevisiae* are phylogenetically closely related [54]. Hence, many orthologous gene functions may have been conserved between the two species. In our study, however, we found several mutants with reduced or increased fitness in rich media corresponding to genes whose inactivation in *S. cerevisiae* resulted in entirely different or opposing phenotypes. These include homologous genes from the RAS-PKA pathway (*RAS2*, *GPA2* and *GPR1*) and genes involved in N-glycosylation and outer chain elongation (*ALG5*, *KTR2* and *MNN4*). Moreover, several genes known to be essential in *S. cerevisiae* were non-essential in *C. glabrata*, although their inactivation often confers reduced fitness or morphology defects. These include *MEC1*, encoding a master DNA damage checkpoint kinase [88], and *CBK1*, although the latter is only essential in the yeast background S288c [89]. *CBK1* encodes a RAM network kinase that is central to the establishment of cell polarity and involved in septum formation and cell separation and morphogenesis [90]–[93]. Notably, inactivation of the orthologous genes in *C. albicans* is not lethal [93]–[95]. Hence, Mec1- and Cbk1-dependent regulatory networks may operate differently between *S. cerevisiae* and *C. glabrata* and, possibly, other hemiascomycetous yeasts. Many biological processes and regulatory networks in fungi have evolved under distinct evolutionary pressures [96], [97]. Because *Candida* spp. have evolved as opportunistic pathogens of mammals, including humans, it is fair to propose that adaptation to the host environment and immunity surveillance may have been driving the evolution of functionally rewired genetic regulatory networks [98].

### Biofilm genes

Deletion strains displaying altered ability to form biofilms include, as expected, several mutants lacking cell wall-related genes, whose absence disturb normal cell wall homeostasis; not surprisingly, these mutants also strongly altered CF susceptibility. Lack of the putative kinase gene *PKH2*/*CAGL0I07513g*, whose orthologue in *S. cerevisiae* encodes a component of the alternative cell wall integrity pathway affected biofilm formation. In *C. glabrata*, *PKH2* has two additional paralogues, *CAGL0G04609g* and *CAGL0K06479g*. While *pkh2* mutants are defective in both biofilm formation and show temperature-defective growth on solid media, lack of the other two putative paralogues leaves biofilm formation and temperature sensitivity unaffected (data not shown). These data suggest that apparent *PKH* kinase homologues in *C. glabrata* have acquired some specialization concerning their roles in sensing cell wall integrity or regulating cell surface homeostasis.

### Antifungal drug sensitivity

We generated a chemical-genetic profile of *C. glabrata* relevant to the understanding of drug susceptibilities *in vitro*. In addition to known genes implicated in azole resistance (*PDR1, CDR1, PDH1*), the profiling for azoles revealed only a small number of novel genes (*YPK1, KTR2*) mediating azole tolerance, with *KTR2* even showing a significant azole-specificity for voriconazole. Our data, as well as data from a wealth of clinical isolates, imply that *C. glabrata* utilizes a limited set of mechanisms to mount azole resistance, with ABC transporter-mediated efflux by Cdr1 and Pdh1 being the most important one, both *in vitro* and *in vivo* [32], [67], [99]–[102]. However, it has been noticed that genomic deletions conferring azole resistance when otherwise mutated or overexpressed do not necessarily change sensitivities in the wild type strain [103], perhaps due to genetic (functional) redundancy or compensatory mechanisms. Thus, we cannot exclude the existence of other mechanisms and genes promoting azole resistance in *C. glabrata*.

The results from our screen, as anticipated, showed overlap with phenotypic screens in *S. cerevisiae* [104] and *C. albicans* [105] regarding sensitivity to CF, which belongs to the family of the fungicidal echinocandin drugs that inhibit the fungal 1,3-β-D-glucan synthase. Out of 38 mutants showing strong alterations in echinocandin sensitivities, 16 correspond to genes whose orthologues in *S. cerevisiae* and in *Candida spp* (at least for *FKS1*) have been linked to CF tolerance [36], [104]–[106]. Hyperresistance to echinocandins can result from mutations in the glucan synthase genes *FKS1* or *FKS2* [35], as well as through the PKC pathway that mediates CF tolerance in *S. cerevisiae* [66] and *C. albicans* [107]. Accordingly, *C. glabrata wsc1*, *slt2*, *mkk1*, *bck1*, *rlm1* and *fks1* mutants, all lacking key genes of this central pathway, are also hypersensitive to CF in our assays. This overlap points to commonalities in the response to CF between the three species, and also serves to validate the phenotypic profiling for echinocandin susceptibility. Remarkably, 28 genes whose deletion resulted in marked hypersensitivity to CF in *C. glabrata* have not previously been associated with CF susceptibility in *S. cerevisiae* or other fungi including *C. albicans* [105]. Some of these apparent differences between species likely relates to difficulties in determining CF hypersensitivity phenotypes, given that some strains (*cdc12*, *ace2*, *fkh2*, *cbk1*) display aberrant cell morphologies or strong growth fitness defects (*kre6*), all of which interfere with IC-50 quantifications. Regardless, our data clearly identify novel genes implicated in CF tolerance, and imply that these genes may be players in as yet undiscovered CF resistance mechanisms.

Interestingly, cells lacking chitin synthesis genes such as *CHS1* were also hypersensitive to the glucan synthase inhibitor, completely consistent with reports from *S. cerevisiae* [105]. Interestingly, recent data from *C. albicans* demonstrate a role for chitin in regulating cell wall susceptibility to CF [108], [109]. Likewise, the absence of calcineurin subunit genes *CNA1* or *CNB1* leads to CF hypersensitivity, indicating that calcineurin signaling is necessary for buffering or compensating cell wall stress, perhaps by affecting *FKS1* transcript levels through the transcription factor Crz1 [35]. In the mould *Aspergillus fumigatus*, a mutation or inhibition of calcineurin enhances the antifungal potency of CF [110]. Thus, calcineurin inhibitors may exert synergistic effects on cell wall mutants in other fungal species. The impact of CF on calcium signaling in *C. glabrata* is further confirmed by the hypersensitivity of mutants lacking *CCH1* and *MID1*, both of which encode stress-induced calcium channel proteins.

In *S. cerevisiae*, the Ypk1-mediated signaling pathway, which is activated by lipid-sensing [111], constitutes an alternative cell integrity signaling pathway connected to the classical PKC pathway [112], [113]. Remarkably, *C. glabrata* cells lacking *YPK1* and *PKH2* are CF-hypersensitive, suggesting that the Ypk1-mediated signaling plays a major role in regulating CF tolerance or cell wall homeostasis in *C. glabrata* (Figure 4, Figure S7). Taken together, these results indicate that *C. glabrata* employs several signaling pathways to respond to CF-induced cell wall damage and the required subsequent cell wall remodeling. It is therefore not unexpected that mutants displaying CF hypersensitivities include genes that affect trafficking of proteins or surface carbohydrate homeostasis, including chitin deposition and biogenesis.

Genes implicated in the susceptibility to both CF and azoles include those encoding the calcineurin subunits Cna1 and Cnb1 and the PKC pathway components Wsc1 and Slt2. These results confirm that both pathways are necessary for a response to both drug classes and are consistent with data in many fungi showing the synergistic action of calcineurin inhibitors and azoles or CF [36], [81], [84], [114], [115]. Comparison of azole-sensitive, AmB-sensitive and CF-sensitive mutants show that only a small number of gene deletion strains are susceptible to all three or to at least two compounds, which is consistent with their distinct mechanism of action. The few deletions conferring sensitivity to all three drugs occur in genes implicated in stress signaling, and include kinases such as Ypk1 and the mRNA-binding protein Ssd1, which is thought to control expression of surface genes in concert with Cbk1 [116]–[120]. Our results are consistent concerning the phenotypes for the corresponding mutants in *S. cerevisiae*. However, the exact molecular function of Ypk1, a mammalian SGK kinase homologue [121], which regulates sphingolipid biosynthesis and cell integrity pathways in *S. cerevisiae*, remains unclear in *C. glabrata*. It is tempting to speculate though that Ypk1 is part of a kinase network implicated in sensing and regulating membrane perturbations, drug sensitivity and lipid-mediated stress signaling [97], [122].

AmB is thought to interfere with normal membrane bilayer function by forming complexes with ergosterol [123]. Indeed, cells deficient in membrane biogenesis or organelle dynamics may show a synthetic fitness loss upon perturbation of the lipid composition, potentially explaining the AmB hypersensitivity of *snf7*, *vps15* and *sla1* mutants. A similar reason may explain the sensitivity of cells lacking *DEP1*, which, in *S. cerevisiae*, regulates transcription of structural phospholipid biosynthesis genes [124]. Notably, the profiling analysis reveals a strong genetic interaction between AmB action and genes implicated in cell wall function. AmB-sensitive strains include mutants lacking *KRE1* and *SAC7*, which encode proteins implicated in glucan homeostasis, as well as *KTR6, KTR2, CWH41*, whose products affect surface protein glycosylation. Remarkably, the latter three mutants show pronounced azole hypersensitivity, demonstrating a direct link between membrane lipid perturbation, cell wall function and antifungal sensitivity.

The hierarchical clustering of stress-induced phenotypes caused by heat, CF, CR and CW, identifies some 106 putative chemical-genetic interactions (Figure 5). We expected to discover distinct patterns for each compound, since CR and CW mainly affect cell wall structure and composition, whereas CF targets Fks1. All compounds strongly activate cell integrity signaling, which together drives cell wall remodeling and regulates surface homeostasis. Accordingly, the CF profile was enriched for genes involved in cell wall organization, signaling and transcriptional regulators, reflecting the activity of CF as an inhibitor of fungal cell wall biosynthesis (Figure 4). We were surprised that only a few genes in the CF cluster overlap with genes associated with CR and CW sensitivity (Figure 4A), most of which are involved in stress and cell wall signaling (*CNA1*, *CNB1*, *WSC1*) or cell wall biogenesis (*KRE1*, *PSA1*, *ANP1*, *MNN10*), indicating that distinct signaling pathways must cooperate to ensure maintenance of a functional cell wall under various adverse conditions.

As expected, our screens for drug susceptibility and cell wall integrity identified *C. glabrata* orthologues of genes implicated in related processes in other fungi. In addition, we identified novel genes whose functions may be specific to *C. glabrata*, since none of them has been associated with drug sensitivity or cell wall homeostasis. Because the phenotypes are in general hypersensitivities, these data suggest that at least some genes may represent feasible targets for drug discovery. The discovery of a large number of deletion mutants affecting CF tolerance expands our knowledge about plausible mechanisms regulating CF sensitivity. For example, the fact that several CF-sensitive mutants are implicated in exocytic delivery of cell wall components such as chitin, glucan or mannan, implies a constant cross-talk of distinct signaling pathways to control proper cell wall remodeling upon CF-induced surface damage. As the number of chemical-genetic and genomic data in baker's yeast [45], [46] is steadily increasing, our data add novel information concerning the function of related genes in an important human fungal pathogen. Hence, these data represent an important contribution towards a better understanding of drug resistance mechanisms, as well as species-specific differences.

These large-scale phenotypic profiling data also demonstrate the power of the *C. glabrata* knock-out collection, which is, in addition to the *C. albicans* and *C. neoformans* collections [50], [53], to the best of our knowledge, among the three largest academic deletion collections for a human fungal pathogen. We anticipate that this library will facilitate studies on virulence factors and other aspects of *C. glabrata* biology. The use of sequence barcodes, which were adopted from the *S. cerevisiae* gene deletion collection [43], allows for the functional analysis of pools of mutants either *in vitro* or *in vivo* [50], [53]. The use of multiple auxotrophic markers, which do not affect *in vivo* dissemination of strains in standard mouse models of fungal virulence [60] will allow for construction of double or triple mutants, and facilitate genetic interaction studies as well as epistasis analysis of pathway architectures. Notably, screens of the collection in different animal models and the understanding of virulence phenotypes (or lack thereof) can be challenging and laborious requiring a very large number of animals, due to the potential impact of fitness defects on growth *in vivo* or possible genetic redundancy. We suggest that interpretation of virulence phenotypes will be aided by the *in vitro* phenotypic analysis presented here, permitting correlation of *in vitro* phenotypes and *in vivo* fitness effects for *C. glabrata*.

Similar to existing *C. albicans* [50] or *C. neoformans* [53] collections, the *C. glabrata* mutant library constitutes a valuable tool for the fungal research community to study the function of virulence and drug resistance genes in *C. glabrata*. In view of rapid changes in the epidemiology of fungal infections, with *C. glabrata* infections showing ever-increasing clinical importance reaching up to 30% prevalence in some countries [33], [125], this work is the first large-scale contribution to the systematic analysis of mechanisms implicated in antifungal drug resistance and *C. glabrata* pathogenicity. Notably, much of the previous work aimed at unraveling the molecular basis of drug resistance mechanisms in *C. glabrata* have been based on what is known from baker's yeast [104], [126] or other Candida pathogens [105]. Although gene synteny has been largely conserved between pathogenic an non-pathogenic yeasts such as *C. glabrata* and *S. cerevisiae*, extensive rewiring of signaling pathways generated distinct and species-specific functions for seemingly orthologous genes. Indeed, our work clearly demonstrates that comparing and predicting drug resistance or virulence phenotypes for *C. glabrata* based on data from even related yeasts requires extensive experimental verification and the use of loss-of-function approaches. This fungal pathogen deletion collection will pave the way for these future efforts.

## Materials and Methods

### Ethics statement

The use of clinical C. glabrata isolates was approved through respective ethics committees according to national regulations.

### Media and growth conditions

YPD (1% yeast extract, 2% peptone, 2% dextrose) media were prepared as described elsewhere [127]. Synthetic complete (SC) medium contained 0,67% YNB, 2% glucose supplemented with 1×CSM (ForMedium; complete synthetic mixture) containing histidine, tryptophane and leucine, which are required for growth of the deletion strains. Plates contained 2% agar.

### Bioinformatic analysis and gene selection

Genes of different functional categories were manually selected based on potential function in virulence and drug resistance. The categories involved genes of signaling pathways, kinases, ABC transporters and permeases, GPI-anchored proteins, cell wall associated genes, genes involved in glycosylation, phospholipid biosynthesis, histone modification, iron metabolism, and several genes with no obvious homologue in *S. cerevisiae*. The genes were selected by their homology to *S. cerevisiae* based on these functional categories (SGD annotations; http://www.yeastgenome.org). *C. glabrata* orthologues of the selected genes were first identified using a BLAST approach. The three best-aligned hits for each gene were saved and the *C. glabrata* homologue with the highest P-value was arbitrarily defined as the *C. glabrata* orthologue of a given gene in baker's yeast and named accordingly. In addition, a complete catalogue of orthology and paralogy relationships between *C. glabrata* genes and their homologues in 16 other fully-sequenced fungi was derived using a phylogenetic approach [128]. For this a complete collection of Maximum Likelihood phylogenetic trees for all *C. glabrata* genes, the so-called phylome, was generated using the automated pipeline described elsewhere [129]. Gene phylogenies, alignments and orthology and paralogy predictions are publicly available through PhylomeDB (http://www.phylomedb.org).

### Primer design and generation

Oligonucleotide sequences for generation of the deletion cassettes and strain verification were automatically designed, using a custom-written Perl script called *PrimerList* (W. Glaser, unpublished data). PrimerList utilizes Bioperl to read and process nucleotide sequences and uses the EMBOSS [130] programs eprimer 3 and stssearch to find suitable primersets. For PCR-based generation of knock-out constructs, upstream and downstream fragments for genomic recombination were chosen to have a size between 450 and 550 nucleotides. 5′5′ (forward) and 3′3′ (reverse) primers were chosen to have a length of 20 to 30 bp, a GC content between 30 and 60% and a melting temperature of 50°C±4°C with a GC clamp. 5′3′, 3′5′ primers were chosen to have a length of 20 to 30 bp plus the 20 bp constant overlap sequence (Figure 1, Table S3, ‘barcode sequence sheet’), a GC content between 30 and 60% and a melting temperature of 50°C±4°C. 5′3′ (reverse), 3′5′ (forward) primers were chosen to bind exactly adjacent to the coding sequence, including the start codon ATG or the stop codon, respectively. 5c and 3c control primers have a length of 20 to 25 bp, a GC content between 40 and 60% and a melting temperature between 50°C to 60°C. The product size of the control PCR is between 750 and 900 bp. Internal control primers (5i and 3i) were designed to bind inside the coding sequence and to give a product of 400 to 500 bp in size. The primers have a length of 20 to 25 bp, a GC content between 40 and 60% and a melting temperature between 55°C to 60°C. Oligonucleotides were commercially purchased in 96-well plate format (Eurogentec, Belgium). Six plates were needed for each set. Each of the six plates (5′5′, 5′3′, 3′5′, 3′3′, 5c, 3c) contained the primers for a specific gene at the exact same well position.

### Generation of gene deletion cassettes by fusion PCR

The dominant marker *NAT1* was amplified from plasmid pJK863 [57] using the primers fp_NAT1-U2 and rp_NAT1-D2 to add the constant 20 bp sequences U2 and D2. The PCR product was ligated into a pGEM-T vector (Promega), generating plasmid pTS50. For the fusion PCR step, deletion cassettes were generated using a modified fusion PCR protocol [51]. Briefly, 500 bp long flanking homology regions were amplified from ATCC2001 genomic DNA with primer pairs 5′5′/5′3′ and 3′5′/3′3′ adding the constant overlap sequence (U1/D1) of 20 bp and purified by ethanol precipitation. The conditions for a 50 µl reaction were as follows: 1× Taq buffer (50 mM KCl, 10 mM Tris-HCl (pH 9.0, 25°C), 0.1% TritionX-100, 1.5 mM MgCl2), 0.2 µM dNTPs, 0.5 µM each primer, 1 µl Taq-Polymerase and genomic wild type DNA from strain ATCC2001; 93°C for 5 minutes, 35 cycles 93°C for 30 s, 45°C for 30 s, 72°C for 90 s, finally 10 minutes at 72°C.

The dominant marker *NAT1* was amplified from plasmid pTS50 in a separate PCR reaction using primers 5M and 3M, adding unique barcode tags and constant complementary U1 and D1 sequences. Marker fragments were gel-purified in 0.7% agarose gels. The conditions for a 50 µl reaction were as follows: 1× Taq buffer, 0.2 µM dNTPs, 0,5 µM each primer, 1 µl Taq-Polymerase and plasmid TS50; 93°C for 3 minutes, 32 cycles 93°C for 30 s, 49°C for 30 s, 72°C for 2,5 minutes, finally 10 minutes at 72°C. Fusion PCR was carried out in a 50 µl volume with the same conditions as above: 1× ExTaq buffer, 0.2 µM dNTPs, 0.5 µM each primer, 0.5 µl ExTaq-Polymerase (TaKaRa) and 3 µl marker fragment, 1.25 µl each flanking homology fragment; 93°C for 3 minutes, 35 cycles 93°C for 30 s, 45°C for 30 s, 72°C for 3 minutes, finally 10 minutes at 72°C. The final deletion construct was purified by ethanol precipitation.

### Complementation of *C. glabrata* deletion mutants

Cloning of *C. glabrata* ORFs in the pDONR207 vector was done as described in [131]. Briefly, for each of the selected ORFs, a forward primer including the *attB1* site and the first 10 codons of the ORF and a reverse primer including the *attB2* site and the last ten codons of the ORF were designed and synthesized at Pasteur-Genopole-Ile-de-France oligonucleotide synthesis platform (Table S13). ORFs were amplified from genomic DNA of *C. glabrata* strain ATCC2001 [54] using Phusion High-Fidelity DNA Polymerase (New England Biolabs) and 30 cycles of amplification with elongation times varying from 1 to 3 min. according to the ORF size. The resulting PCR products were checked by agarose gel electrophoresis, ethanol precipitated and, following resuspension in Tris-EDTA, mixed with the donor plasmid pDONR207 (Invitrogen), and subjected to a recombination reaction with Invitrogen Gateway BP Clonase. The recombination mixes were transformed into *E. coli* strain DH5α and one transformant per ORF was used for plasmid preparation. The cloned ORFs were sequenced from the 5′- and 3′-ends using Sanger sequencing.

To construct a replicative, Gateway-compatible *C. glabrata* expression vector, the *C. glabrata TDH3* promoter amplified from genomic DNA of *C. glabrata* strain ATCC2001 using oligos CgTDH3p-fwd (5′-GCGCCCGGTACCCAGGTGATCATATCACTCACA-3′) and CgTDH3p-rev (5′-GGGCCGACTAGTGTTATGTTTGTTGTGATTTGTA-3′), and a Gateway cassette amplified from plasmid CIp10-P_TET_-GTW [132] were cloned into the replicative vector pCgACT-14 [133], yielding the Destination vector pCgACT-P_TDH3_-GTW. Transfer of *C. glabrata* ORFs and the GFP ORF from pDONR207 into pCgACT-P_TDH3_-GTW was as described in [131]. An aliquot of each Entry plasmid was mixed with 50 ng of the Destination plasmid and subjected to a recombination reaction with Invitrogen Gateway LR Clonase. The recombination mixes were transformed into *E. coli* strain DH5α and one transformant was used for plasmid preparation. *Eco*RV digestion was used to verify the cloning of the appropriate ORF. A list of the plasmids used in this study is shown in Table S11. The expression plasmids were transformed into the corresponding *C. glabrata* deletion strain according to [134]. Transformants were selected for prototrophy. The resulting strains are listed in Table S12.

### Transformation of *C. glabrata* by electroporation

For transformation of the background strain HTL, we used a modified electroporation protocol [56]. Aliquots of 50 ml of a *C. glabrata* culture in YPD at an optical density of 600 nm (OD_600_) of 1.3 were harvested, washed with H_2_O, resuspended in 1× TE buffer, 100 mM LiAc and incubated at 30°C for 30 minutes with slow shaking (130×rpm). After addition of 250 µl 1M DTT and further incubation at 30°C for 60 minutes (130×rpm), 40 ml of H_2_O were added and the cells were harvested at 1000 g for 5 minutes at 4°C. The cells were washed with 25 ml H_2_O, subsequently with 5 ml 1 M cold sorbitol, finally resuspended in 550 µl 1 M sorbitol and kept on ice until use. Sterile electroporation cuvettes were precooled on ice and loaded with a mix of 40–45 µl electrocompetent cells and 5–10 µl linear DNA deletion construct (app. 2–3 µg DNA). Cells were left on ice for 10 minutes and electroporation was carried out with a BioRad GenePulser (200Ω, 1.5 kV, 25 µF). For recovery, 950 µl YPD was added and cells incubated for 4 h shaking at 30°C, before plating on YPD supplemented with 200 µg/ml Nourseothricin (Werner Bioagents, Jena). The plates were incubated for 48 hours at 30°C. For auxotrophic marker constructs the cells were recovered for 1 h at 30°C before plating on selective SC medium. Transformants were patched on YPD/Nourseothricin plates for colony PCR. For 96-well parallel electroporation, 300 ml of culture were grown to OD_600_ of 1.3, split into 50 ml aliquots and treated as described above. For electroporation, we used a BTX Harvard Apparatus ECM630 electroporation device with a HT-100 plate handler.

### Verification by yeast colony PCR

Strains were verified by colony PCR to confirm correct genomic integration of the deletion cassette, as well as loss of the wild type allele according to the following protocol (Figure 1B). Transformants were patched on selective plates and incubated at 30°C for 24 h. Cells were resuspended in 40 µl PCR mix 1 (0.2 µM dNTPs, 0.5 µM of each gene specific primer 5c/3c up-/downstream of the homology region and marker specific primer 5M/3M) and heated for 10 minutes at 93°C. After cooling on ice, 10 µl polymerase mix (5 µl 10× PCR buffer and 1 µl Taq-Pol.) were added per reaction and a regular PCR was performed (93°C for 5 min, 25 cycles 93°C for 30 s, 45°C for 30 s, 72°C for 90 s, final 10 min 72°C). To verify the loss of the coding sequence (CDS), colony PCR was essentially performed the same way. Oligonucleotides used to screen for CDS loss bind inside the CDS to generate a product of 500 bp. All internal primers were also checked for functionality in a separate PCR reaction, amplifying the fragment from genomic wild type DNA.

### Phenotypic profiling

For phenotypic analysis of the deletion collection, mutant cells were re-streaked from frozen stocks and grown for 48 h at 30°C on fresh YPD plates. Each of the three plates containing independent transformants of the same set of genes was arrayed into a 384-spot format serving as source plates. Phenotypic profiling of the deletion collection was performed using a robot on YPD plates (RoToR HDA, Singer Ltd., Roadwater, UK) or by manually spotting (two 1∶10 dilutions from 24 h culture in SC) on SC plates supplemented with the compounds to be tested. Unless otherwise indicated, we added 120 ng/ml CF, 50 µg/ml CW (Sigma-Aldrich) or 250 µg/ml CR (Sigma-Aldrich) as supplements to media from sterile stock solutions after autoclaving. Plates were routinely incubated at 30°C for up to 3 days and scanned photographed with S&P Imaging system (S&P Imaging, Canada), after 24, 48 and 72 h for documentation. Primary hits were manually rescreened for confirmation in 1∶10 serial dilutions. Exponentially growing cells were adjusted to an OD_600_ of 0.1. Equal volumes of serial dilutions (1∶10, 1∶100 and 1∶1000) were spotted on YPD plates containing drugs and incubated as described above.Hypersensitive mutants identified as primary hits by robotic screening were independently re-screened manually in 96-well format, to verify growth phenotypes on agar plates containing various concentrations of xenobiotics. All manual re-screening assays were carried out independently at least in biological triplicates in four different laboratories, including the confirmation of hits by serial-dilution spot assays on agar plates or as appropriate by microdilution assays in liquid cultures.

### Azole and drug susceptibility screenings

Azole susceptibility screenings were carried out by a modified endpoint method [135] in liquid culture in microtiter plates, using the following drug concentrations: 4 µg/ml Fluconazole, 0.1 µg/ml Voriconazole, (all azoles from Discovery Fine Chemicals), 3 µg/ml AmB (Discovery Fine Chemicals). Cells were grown overnight in deep well plates to stationary phase, diluted 100-fold in sterile water and 100 µl suspension mixed with 100 µl of 2× YPD containing a 2× drug concentration (app. 10^5^ cells/well). After incubation at 30°C for 24 h and 48 h, cells were resuspended and the OD_600_ was measured with a Victor plate reader (Perkin Elmer, USA).

### Microbroth dilution assay for IC50 determination

To determine the IC_50_ of antifungal drugs a modified protocol of the microbroth dilution assay was used [136]. Briefly, an overnight culture was diluted 1∶100 in YPD, regrown to an OD_600_ of 1 and an inoculum of 2.5×10^4^ cells/ml was prepared. Antifungal stock solutions were prepared in DMSO. Two fold serial dilutions of the drugs were then prepared in water in a deep well plate and stored at −20°C until use. 100 µl of two fold serial drug dilutions were distributed in triplicates into a flat bottom microtiter plate. The last wells free of antifungal drugs served as a growth control. After adding 100 µl of the inoculum (200 µl total volume), plates were incubated at 30°C for 24 h and 48 h in a humid environment to avoid evaporation. OD_600_ was determined with a plate reader. Endpoint readings were set as the antifungal concentrations, causing at least 90% growth inhibition after 24 h of growth when compared to the control. The IC_50_ was determined by linear regression using Graph Pad Prism software.

### Fitness analysis

Growth curves were performed in 96-well plates in a Tecan Infinite M200 microplate reader or a Tecan Sunrise microplate reader. *C. glabrata* strains were grown in YPD at 30°C. For each 96-well plate, the doubling times of each of the 96 tested strains were calculated based on the time necessary for a given strain to go from OD 0.15 to OD 0.6. The relative fitness of a strain was then calculated as the ratio of the average doubling time of all strains within the third to eighth deciles to the doubling time of the strain of interest [68]. Means and standard deviations are presented for fitness values determined for one or two independent knock-out mutants in two biological replicates. Strains with standard deviation above 0.1 or an absolute difference between the relative fitness of two independent knock-out mutants above 0.1 were not considered further. In order to identify among the remaining strains those that showed significantly increased or decreased fitness, the average and standard deviation of the fitness for strains within the second to ninth deciles were calculated. Strains were classified based on the number of standard deviation between their fitness and the average fitness. Strains with decreased fitness had a fitness at least two standard deviations below the average fitness. Strains with increased fitness had a fitness at least two standard deviations above the average fitness.

### Biofilm formation assays

Biofilms were produced in 96-well plates as previously described [75]. Briefly, saturated cultures in YPD were pin-inoculated diluted in 100 µl SD 0.4% glucose medium in 96-well polystyrene plates and incubated at 37°C for 24 h. The 96-well plates were then washed with PBS using a HydroFlex platform (Tecan) and 100 µl of a 1× FDA solution (50× stock: fluorescein diacetate, 2 g l^−1^ in acetone; diluted to 1× in PBS) was added per well [74]. Plates were wrapped in aluminium foil and incubated for 1 h at 37°C before measuring fluorescence in a Tecan Infinite M200 microplate reader using an excitation filter of 486±9 nm and an emission filter of 535±20 nm. The relative biofilm fitness of a strain was then calculated as the ratio of the OD_535 nm_ recording for the strain of interest to the average of the OD_535 nm_ recordings obtained for strains within the third to eighth deciles of all OD_535 nm_ recordings obtained within the 96-well plate to which the strain of interest belonged. Means and standard deviations are presented for fitness values determined for one or two independent knock-out mutants in two biological replicates. Strains with standard deviation above 0.3 or an absolute difference between the 2–4 relative biofilm fitness values above 0.5 were not considered further. In order to identify among the remaining strains those that showed significantly increased or decreased ability to form biofilm, the average and standard deviation of the relative biofilm fitness for strains within the second to ninth deciles were calculated. Strains were classified based on the number of standard deviation between their biofilm fitness and the average biofilm fitness. Strains with decreased biofilm fitness had a relative biofilm fitness at least two standard deviations below the average relative biofilm fitness. Strains with increased biofilm fitness had a relative biofilm fitness at least two standard deviations above the average fitness. Strains with decreased or increased biofilm fitness were further evaluated by performing a biofilm assay in quadruplicate for each of two independent isolates. Data obtained for the 58 candidate strains were compared to those obtained for wild type isolates (96 independent values) using the Wilcoxon test. A p value below 0.01 was considered as indicative of a significant difference with the wild type strain. In this assay, a *yak1Δ* mutant was found significantly impaired in biofilm formation while a *sir3Δ* mutant showed significantly elevated biofilm production as previously shown [75].

## Supporting Information

Figure S1 **Generation of the new triple auxotrophic strain HTL.** (A) Cloning strategy using the *SAT1* flipper for recyclable deletion cassettes of *C. glabrata TRP1*, *LEU2* and *HIS3* genes. 500 bp homology flanking regions were ligated into ApaI/XhoI and SacII/SacI restriction sites in pSFS2a, the deletion cassette excised with ApaI/SacI and the resulting fragment used to transform *C. glabrata* ATCC2001. (B) Growth of ATCC2001 wild type strain, HTL and HTL reference strain on YPD, supplemented with 200 µg/ml Nourseothricin and SC plates lacking histidine, leucine or tryptophan. (C) Growth of ATCC2001 wild type, HTL and HTL reference strains at 30°C and 37°C on solid YPD and minimal medium (YNB, ammonium sulfate, glucose, histidine, leucine or tryptophan), as well as in liquid YPD at 30°C and 37°C.
(TIF)

Figure S2 **Verification of** ***C. glabrata*** **sensitivities to antifungals and hyperosmolarity.** Deletions were tested for (A) osmostress (NaCl), (B) fluconazole (Flc) and (C) CF susceptibility. Serial dilutions of *C. glabrata* ATCC 2001, HTL and selected deletion strains were spotted onto YPD plates supplemented with the indicated compounds. Plates were incubated for two days at 30°C.
(TIF)

Figure S3 **Sensitivity of** ***C. glabrata*** **strains to NaCl treatment.** Confirmation of NaCl sensitivities of osmosensitivity mutants on agar plates. Deletions strains were spotted in serial dilutions on synthetic medium supplemented with 1M NaCl and growth was monitored over 3 days at 30°C.
(TIF)

Figure S4 **Deletions with altered fitness under planktonic or biofilm growth conditions.** VENN diagrams showing the overlap between planktonic and biofilm growth. (A) 36 strains showed a decrease in fitness in biofilm conditions, while 55 strains show a fitness decrease in planktonic conditions. 13 strains showed a decrease in fitness under both conditions. (B) 36 mutants displayed a fitness gain under biofilm conditions, while 35 mutants showed increased fitness under planktonic conditions. 8 strains showed an increase in both growth conditions. SD (standard deviation), Fb (fitness biofilm growth), F (fitness planktonic growth).
(TIF)

Figure S5 **Susceptibilities of** ***C. glabrata*** **mutants to azoles.** Susceptible strains identified in the primary screenings were subjected to IC_50_ determination. Overnight cultures were grown to an OD_600_ of 1.0 and a microdilution assay was carried out in YPD to determine IC_50_ values as described in Materials & Methods. The OD_600_ was determined in a microplate reader after 24 h and 48 h of incubation at 30°C. Each strain was tested in triplicates and mean values normalized to the untreated control were plotted against the antifungal concentration of fluconazole (A) and voriconazole (C). The IC_50_ was calculated by nonlinear regression (curve fit), using GraphPad Prism for fluconazole (B) and voriconazole (D). Shown are data of 24 h measurements. Bars denote standard deviations.
(TIF)

Figure S6 **Susceptibilities of** ***C. glabrata*** **mutants to AmB.** AmB-susceptible strains identified in the primary robotic screening were subjected to IC_50_ determination. Overnight cultures were grown to an OD_600_ of 1.0 and a microdilution assay carried out in YPD to determine IC_50_ for AmB (A) as described in Materials & Methods. The OD_600_ was determined in a microplate reader after 24 h and 48 h of incubation at 30°C. Each strain was tested in triplicates and mean values normalized to the untreated control were plotted against the antifungal concentration. The IC_50_ were calculated for each mutant by nonlinear regression (curve fit), using GraphPad Prism (B). Shown are data of 24 h measurement. Bars indicate standard deviations.
(TIF)

Figure S7 **Sensitivity of** ***C. glabrata*** **deletion strains to cell integrity stressors.** Deletion strain sensitivities were tested against CF, CW, CR, and 42°C in liquid culture and on plates. (A) Growth susceptibilities of *C. glabrata* strains to CF. Susceptible strains identified in the primary robotic screenings were subjected to IC_50_ determination. Overnight cultures were grown to OD_600_ of 1.0 and a microdilution assay was carried out in YPD to determine IC_50_ as described in Materials & Methods. The optical density (OD) of each well was determined at 600 nm on a microplate reader after 24 h and 48 h of incubation at 30°C. Each strain was tested in triplicates and mean values normalized to the untreated control were plotted against the antifungal concentration. The IC_50_ was calculated by nonlinear regression (curve fit), using GraphPad Prism. Shown are data of 48 h measurement. Bars denote standard deviations. (B) Confirmation of CF sensitivity on plates. Strains were spotted in serial dilutions on synthetic medium supplemented with 120 ng/ml caspofungin (CF) and growth was monitored over 3 days at 30°C. (C) Congo Red (CR), heat and Calcofluor White (CW) sensitivity on plates. Strains were spotted in serial dilutions on synthetic medium supplemented with 250 µg/ml Congo Red (CR) or 50 µg/ml CW and growth was monitored over 3 days at 30°C. For heat stress assay cells were grown at 42°C over three days.
(TIF)

Figure S8 **Caspofungin and azole sensitivities of** ***C. glabrata*** **deletions in different genetic backgrounds.** Sensitivities of deletion strains constructed in the HTL (a) and the *his3* (b) background strains were tested for caspofungin (CF), fluconazole (Flc) and voriconazole (Vor) susceptibility on plates. Strains were spotted in serial dilutions on synthetic medium supplemented with the indicated drug concentrations, and growth monitored for 3 days at 30°C. Screening was performed for (A) *RPD3*, (B) *CRZ1*, (C) *RLM1* and (D) *PDR1*.
(TIF)

Figure S9 **Caspofungin sensitivity of** ***C. glabrata*** **revertant strains.** Sensitivities of deletion strains carrying the corresponding wild type gene or a control plasmid containing GFP were tested for caspofungin (CF) susceptibilities on plates. Strains were spotted in serial dilutions on synthetic medium supplemented with CF and growth was monitored over 3 days at 30°C. HTL GFP: HTL control strain carrying GFP-containing plasmid;
(TIF)

Table S1 **Classification of functional gene categories.** Genes were selected by their homology to *S. cerevisiae* based on SGD gene ontology annotations (http://www.yeastgenome.org). *C. glabrata* orthologues were identified using a BLAST approach. The three best-aligned hits for each gene were saved according to decreasing P values. The orthologues have been remapped using a tree-based approach applying the algorithm described in [50], [53]. A total number of 1047 *C. glabrata* genes were subjected to gene disruption.
(DOC)

Table S2 ***C. glabrata*** **background recipient strains used in this study.** The triple auxotrophic strain HTL and isogenic single and double deletion strains were generated in the sequenced reference strain ATCC2001. Strain HTL was used as recipient strain for the generation of the deletion mutants in the strain library. All other strains of the deletion strain collection are deposited in the *C. glabrata* deletion strain library (http://funpath.cdl.univie.ac.at) and listed in Table S3.
(DOC)

Table S3 **Summary of auxotrophic** ***C. glabrata*** **deletion strains generated in the HTL and** ***his3*** **backgrounds derived from the clinical isolate strain ATCC2001.** Sheet 1: deletion strains + corresponding barcodes. Independent transformants of all generated gene deletion strains are listed with corresponding UP and DOWN tags, position in storage plates, number corresponding to the individual biological replicate (IBR#) for a targeted gene (e.g. the second of three independent replicates for CAGL0A01133g has IBR #2) and the nourseothricin marker used. All data can be searched online under: http://funpath.cdl.univie.ac.at. (Sheet 2: barcode sequences only). Bar-codes used as unique genomic tags in *C. glabrata* deletion strains. 2×96 upstream and downstream barcodes were used for every set of 96 gene deletion strains. These can be amplified from constant regions (U1+U2; D1+D2) identical in every deletion strain. Strains labeled with BM or BG carry individual barcodes and were made in the histidine auxotrophic strain derived from ATCC2001.The reference strain in the HTL background carries a unique barcode not used for any other strain. Barcode sequences were adopted from the systematic *S. cerevisiae* gene deletion project (http://www-sequence.stanford.edu/group/yeast_deletion_project/deletions3.html).
(XLS)

Table S4 **Summary of the phenotypic screening results of the** ***C. glabrata*** **deletion collection.** The table shows all deletion mutants obtained and their corresponding phenotypes. For heat stress, CF, CR, CW and NaCl data the degree of growth inhibition was scored from 5 (wild type) to 0 (no growth). For azole and AmB the phenotypes were classified from 0 (wild type) to 3 (severe defect).
(XLSX)

Table S5 **Fitness raw data of** ***C. glabrata*** **deletion strains with strong variations in relative fitness.** Deletions displaying strong variations between independent mutants in the same gene or independent cultures, identifying 57 strains that were excluded from fitness analysis due to a standard deviation of more than 0.1, or an absolute difference in fitness of more than 0.1 between two biological replicates.
(XLS)

Table S6 **Fitness raw data of** ***C. glabrata*** **deletion mutants.** The table shows the raw data for fitness analysis, including fitness and standard deviation (SD).The orange (>3 SD) and red (>2<3 SD) color-coding indicates genes with deviations below and above the average fitness indices. Grey font indicates mutants where only a single isolate was obtained.
(XLS)

Table S7 **Fitness distribution of** ***C. glabrata*** **knock-out mutants.** The Table shows the distribution of fitness phenotypes. Strains with a standard deviation (SD) above 0.1 or an absolute difference in fitness of more than 0.1 between two biological replicates were excluded from detailed analysis. Classification was based on the number of standard deviations between the fitness of a single strain and the average fitness. A total of 504 deletion strains were scored.
(DOC)

Table S8 **Biofilm raw data - strains with strong variations in relative biofilm fitness.** Some 158 deletion strains were excluded from biofilm analysis due to a standard deviation (SD) of more than 0.3 or an absolute difference of more than 0.5 between replicates.
(XLS)

Table S9 **Biomass quantification using fluorescein diacetate assay.** The table shows the raw data for biofilm analysis, including fitness and standard deviation (SD). Grey font indicates mutants where only a single isolate was obtained. Strains were reliable fitness determination at 30°C was not possible are indicated (Pb plankto). The Planktonic (37°C) column summarizes the results of liquid growth measurements at 37°C (Pb indicates altered growth rate).
(XLS)

Table S10 **Biofilm fitness distribution of** ***C. glabrata*** **knock-out mutants.** Summary of biofilm fitness phenotypes. Strains with a standard deviation (SD) above 0.3, or an absolute difference in biofilm fitness of more than 0.5 between replicates were excluded. Classification was based on the number of standard deviations between the fitness of a single strain and the average fitness. A total of 420 deletion strains were subjected to biofilm fitness analysis.
(DOC)

Table S11 **Plasmids used in this study.**
(DOC)

Table S12 **Revertant strains used in this study.**
(DOC)

Table S13 **Oligonucleotides used for construction of revertant strains in this study.**
(DOCX)
