# Supplementary figures and images for: Systematic Phenotyping of a Large-Scale Candida glabrata Deletion Collection Reveals Novel Antifungal Tolerance Genes
Source: PLoS Pathog. 2014 Jun 19;10(6):e1004211. doi: 10.1371/journal.ppat.1004211 (PMC4063973; doi:10.1371/journal.ppat.1004211)

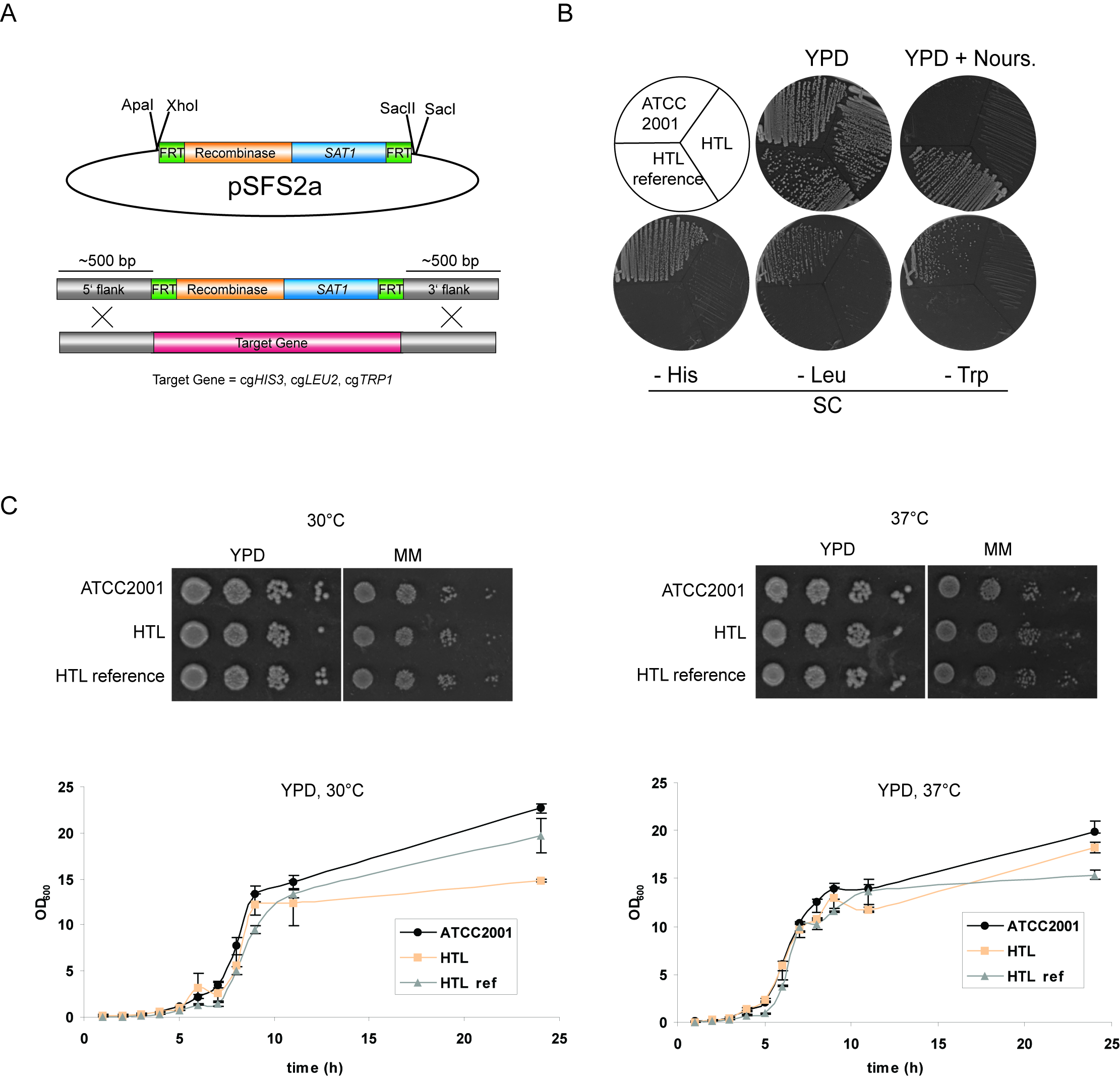

Supplement: Figure S1 — Generation of the new triple auxotrophic strain HTL. (A) Cloning strategy using the SAT1 flipper for recyclable deletion cassettes of C. glabrata TRP1, LEU2 and HIS3 genes. 500 bp homology flanking regions were ligated into ApaI/XhoI and SacII/SacI restriction sites in pSFS2a, the deletion cassette excised with ApaI/SacI and the resulting fragment used to transform C. glabrata ATCC2001. (B) Growth of ATCC2001 wild type strain, HTL and HTL reference strain on YPD, supplemented with 200 µg/ml Nourseothricin and SC plates lacking histidine, leucine or tryptophan. (C) Growth of ATCC2001 wild type, HTL and HTL reference strains at 30°C and 37°C on solid YPD and minimal medium (YNB, ammonium sulfate, glucose, histidine, leucine or tryptophan), as well as in liquid YPD at 30°C and 37°C. (TIF) [file ppat.1004211.s001.tif]

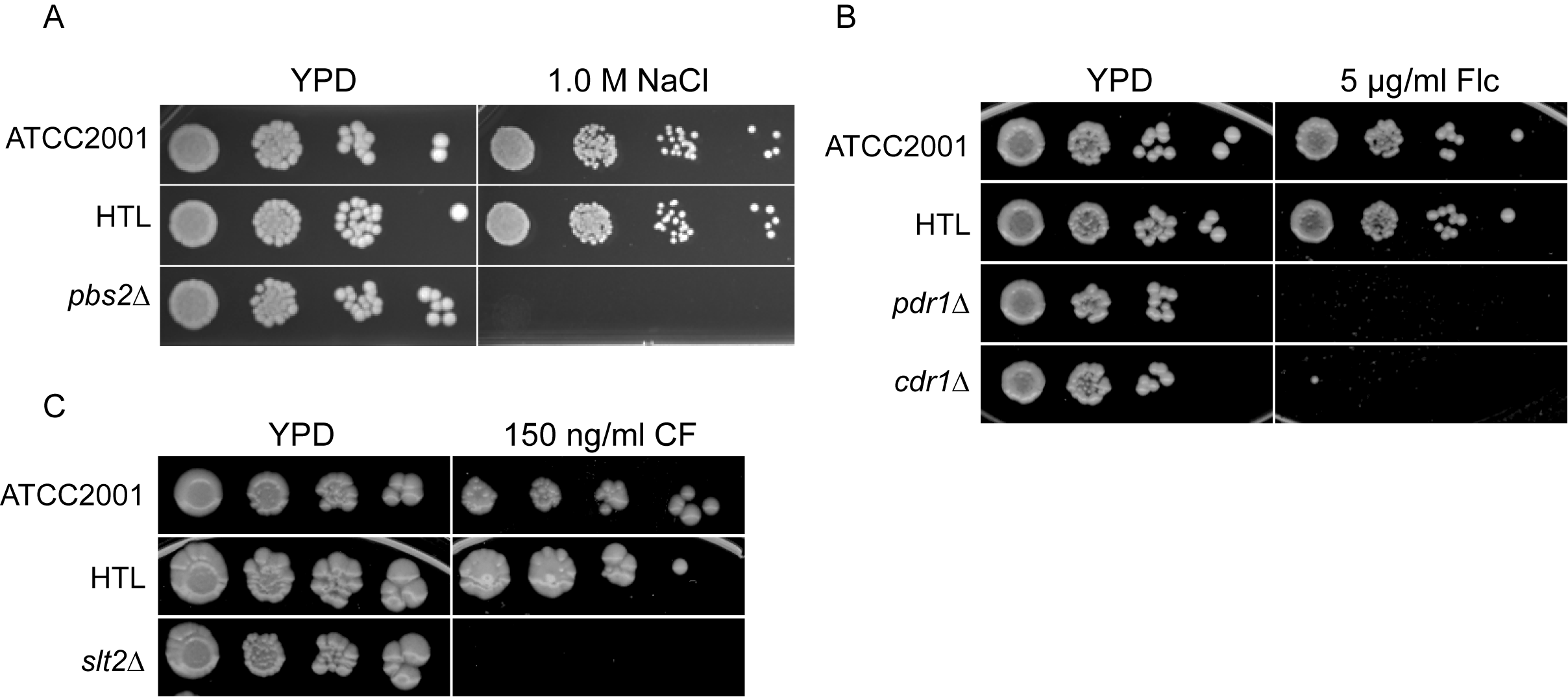

Supplement: Figure S2 — Verification of C. glabrata sensitivities to antifungals and hyperosmolarity. Deletions were tested for (A) osmostress (NaCl), (B) fluconazole (Flc) and (C) CF susceptibility. Serial dilutions of C. glabrata ATCC 2001, HTL and selected deletion strains were spotted onto YPD plates supplemented with the indicated compounds. Plates were incubated for two days at 30°C. (TIF) [file ppat.1004211.s002.tif]

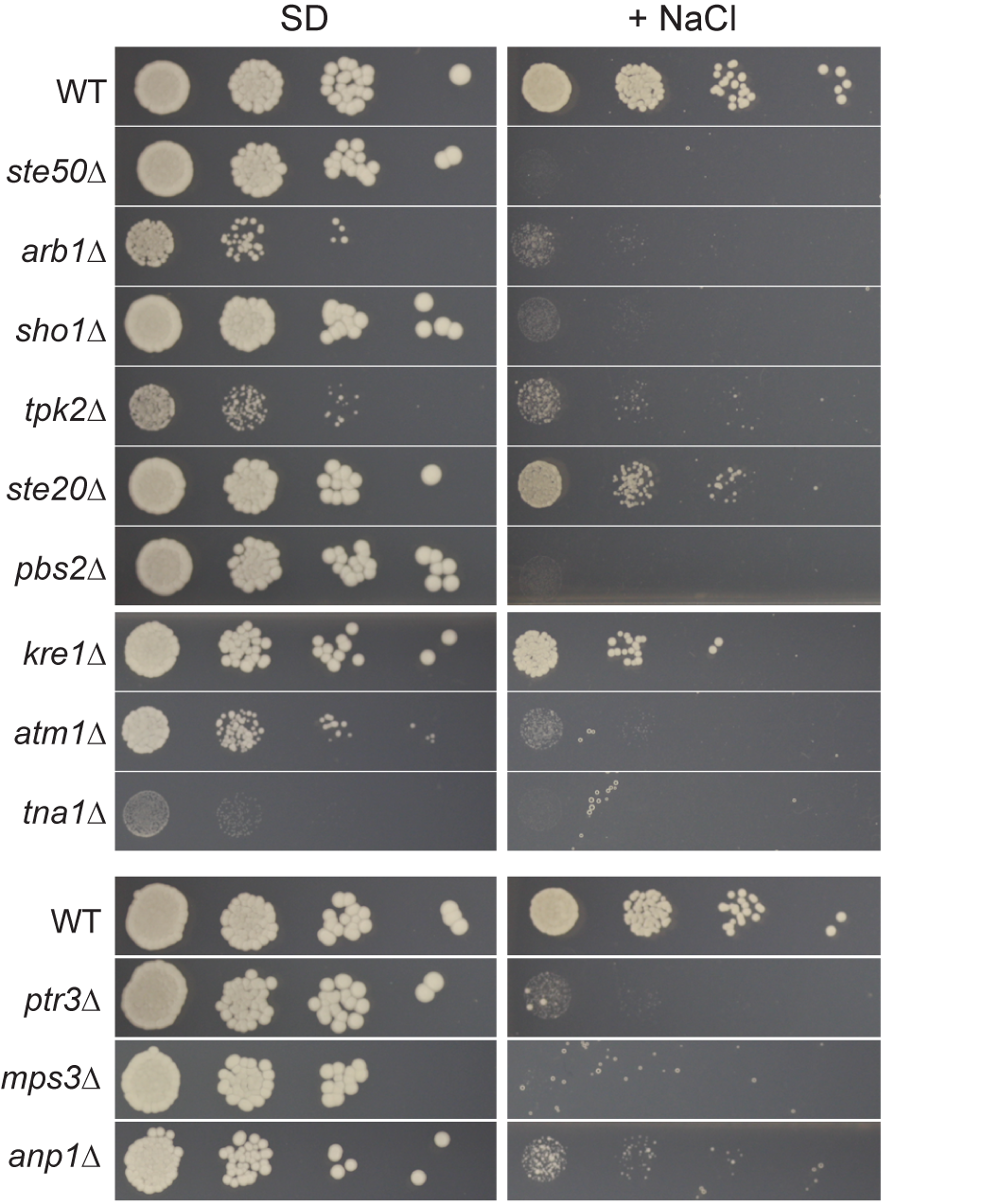

Supplement: Figure S3 — Sensitivity of C. glabrata strains to NaCl treatment. Confirmation of NaCl sensitivities of osmosensitivity mutants on agar plates. Deletions strains were spotted in serial dilutions on synthetic medium supplemented with 1M NaCl and growth was monitored over 3 days at 30°C. (TIF) [file ppat.1004211.s003.tif]

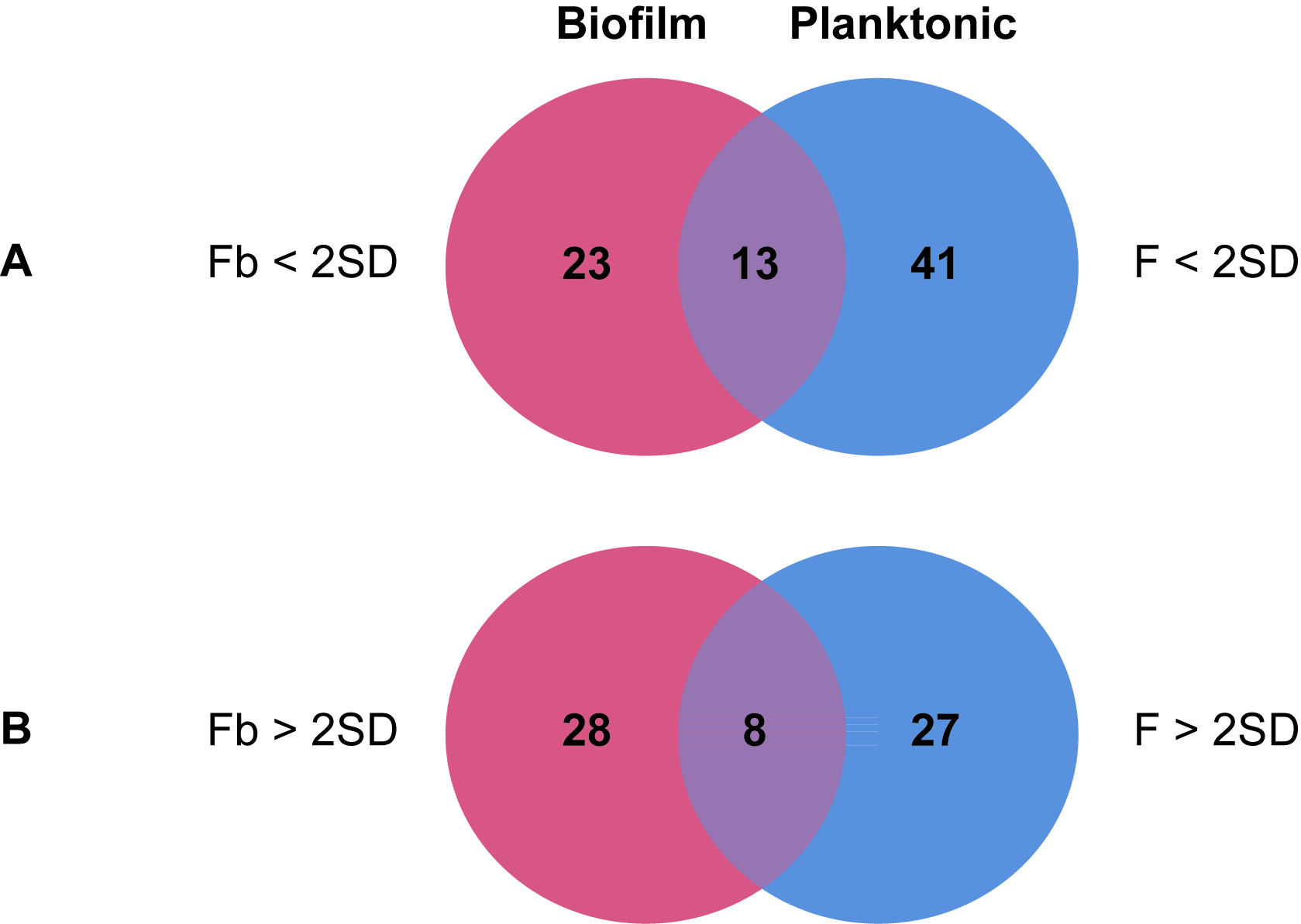

Supplement: Figure S4 — Deletions with altered fitness under planktonic or biofilm growth conditions. VENN diagrams showing the overlap between planktonic and biofilm growth. (A) 36 strains showed a decrease in fitness in biofilm conditions, while 55 strains show a fitness decrease in planktonic conditions. 13 strains showed a decrease in fitness under both conditions. (B) 36 mutants displayed a fitness gain under biofilm conditions, while 35 mutants showed increased fitness under planktonic conditions. 8 strains showed an increase in both growth conditions. SD (standard deviation), Fb (fitness biofilm growth), F (fitness planktonic growth). (TIF) [file ppat.1004211.s004.tif]

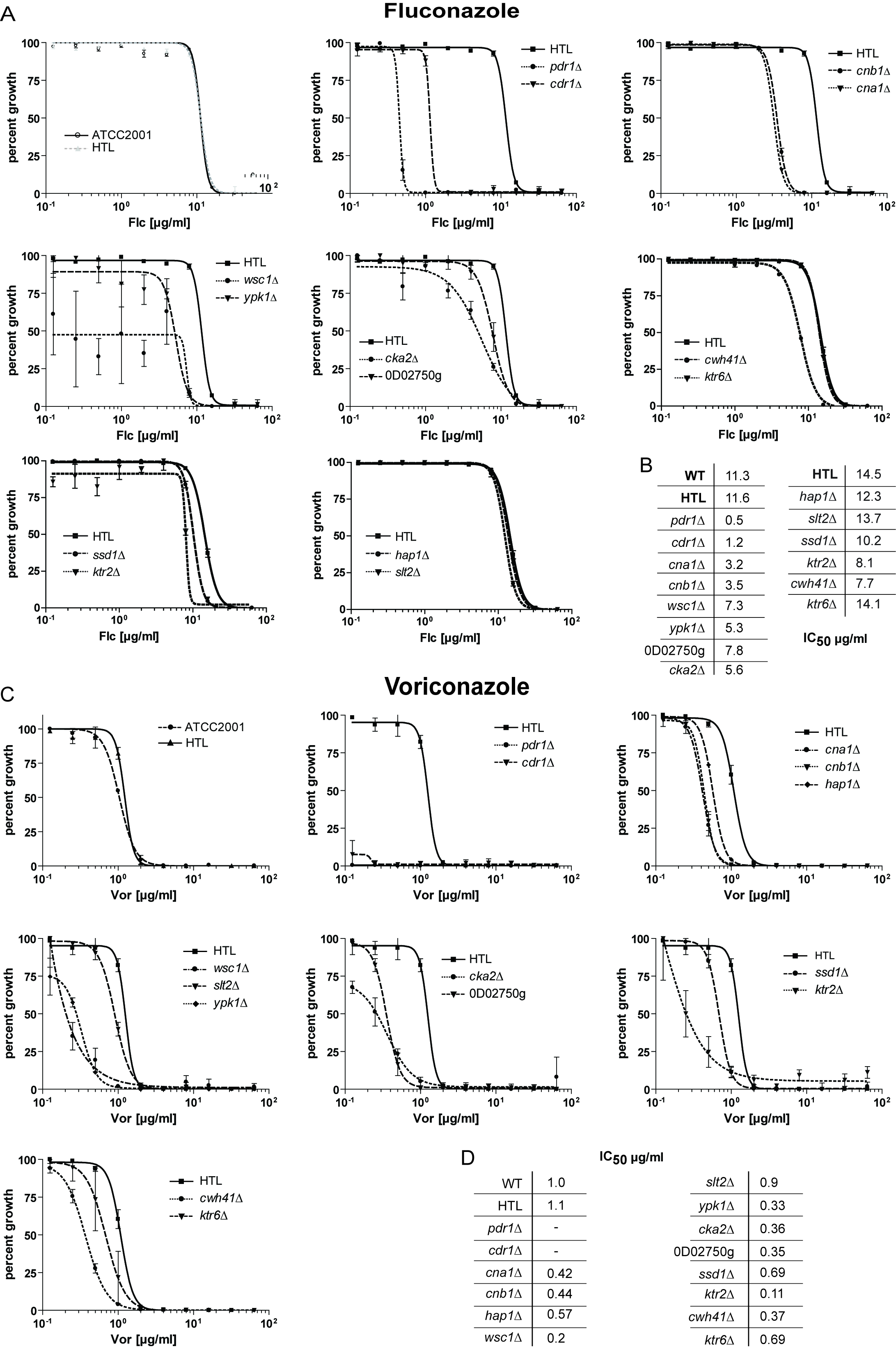

Supplement: Figure S5 — Susceptibilities of C. glabrata mutants to azoles. Susceptible strains identified in the primary screenings were subjected to IC50 determination. Overnight cultures were grown to an OD600 of 1.0 and a microdilution assay was carried out in YPD to determine IC50 values as described in Materials & Methods. The OD600 was determined in a microplate reader after 24 h and 48 h of incubation at 30°C. Each strain was tested in triplicates and mean values normalized to the untreated control were plotted against the antifungal concentration of fluconazole (A) and voriconazole (C). The IC50 was calculated by nonlinear regression (curve fit), using GraphPad Prism for fluconazole (B) and voriconazole (D). Shown are data of 24 h measurements. Bars denote standard deviations. (TIF) [file ppat.1004211.s005.tif]

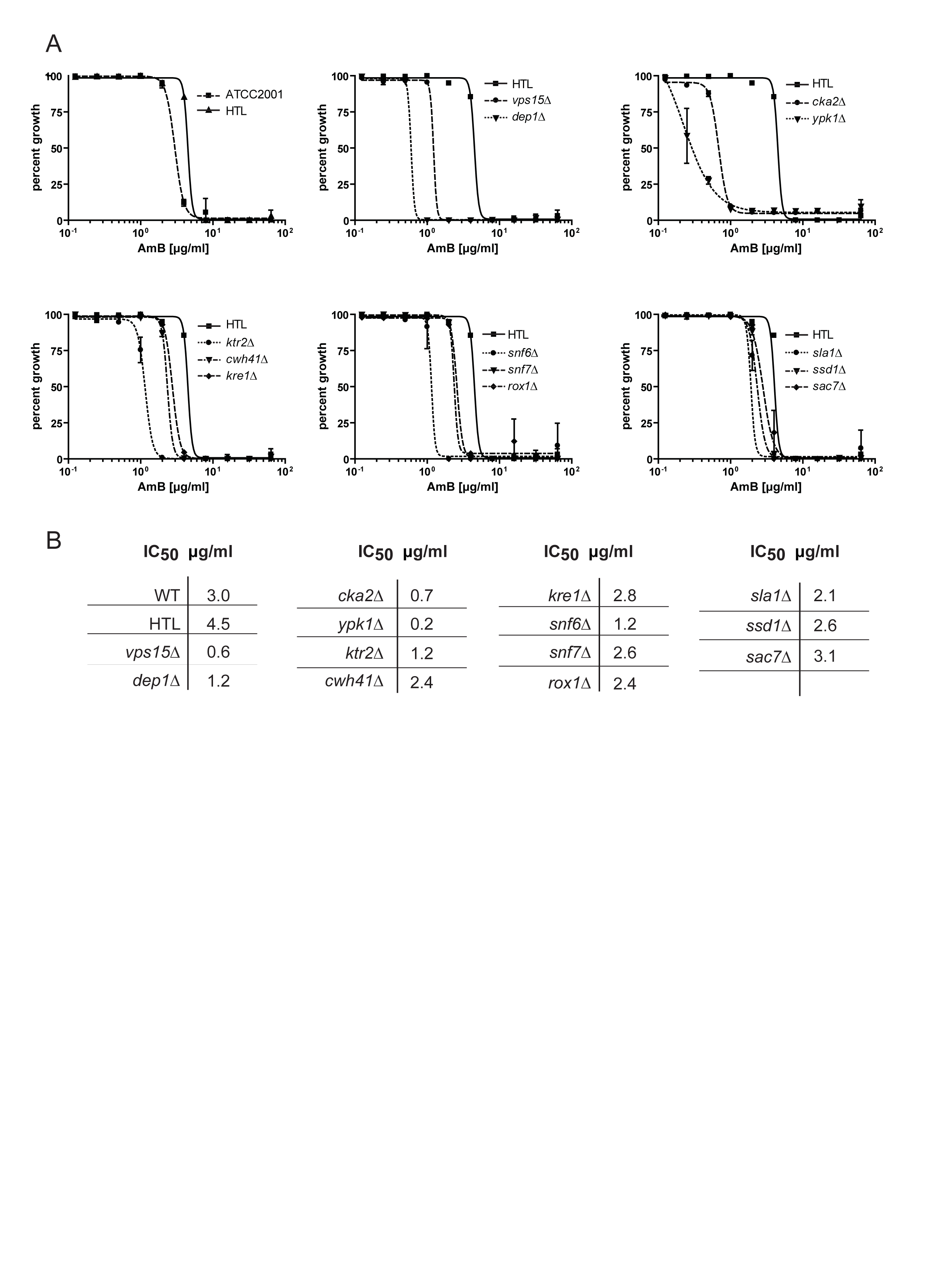

Supplement: Figure S6 — Susceptibilities of C. glabrata mutants to AmB. AmB-susceptible strains identified in the primary robotic screening were subjected to IC50 determination. Overnight cultures were grown to an OD600 of 1.0 and a microdilution assay carried out in YPD to determine IC50 for AmB (A) as described in Materials & Methods. The OD600 was determined in a microplate reader after 24 h and 48 h of incubation at 30°C. Each strain was tested in triplicates and mean values normalized to the untreated control were plotted against the antifungal concentration. The IC50 were calculated for each mutant by nonlinear regression (curve fit), using GraphPad Prism (B). Shown are data of 24 h measurement. Bars indicate standard deviations. (TIF) [file ppat.1004211.s006.tif]

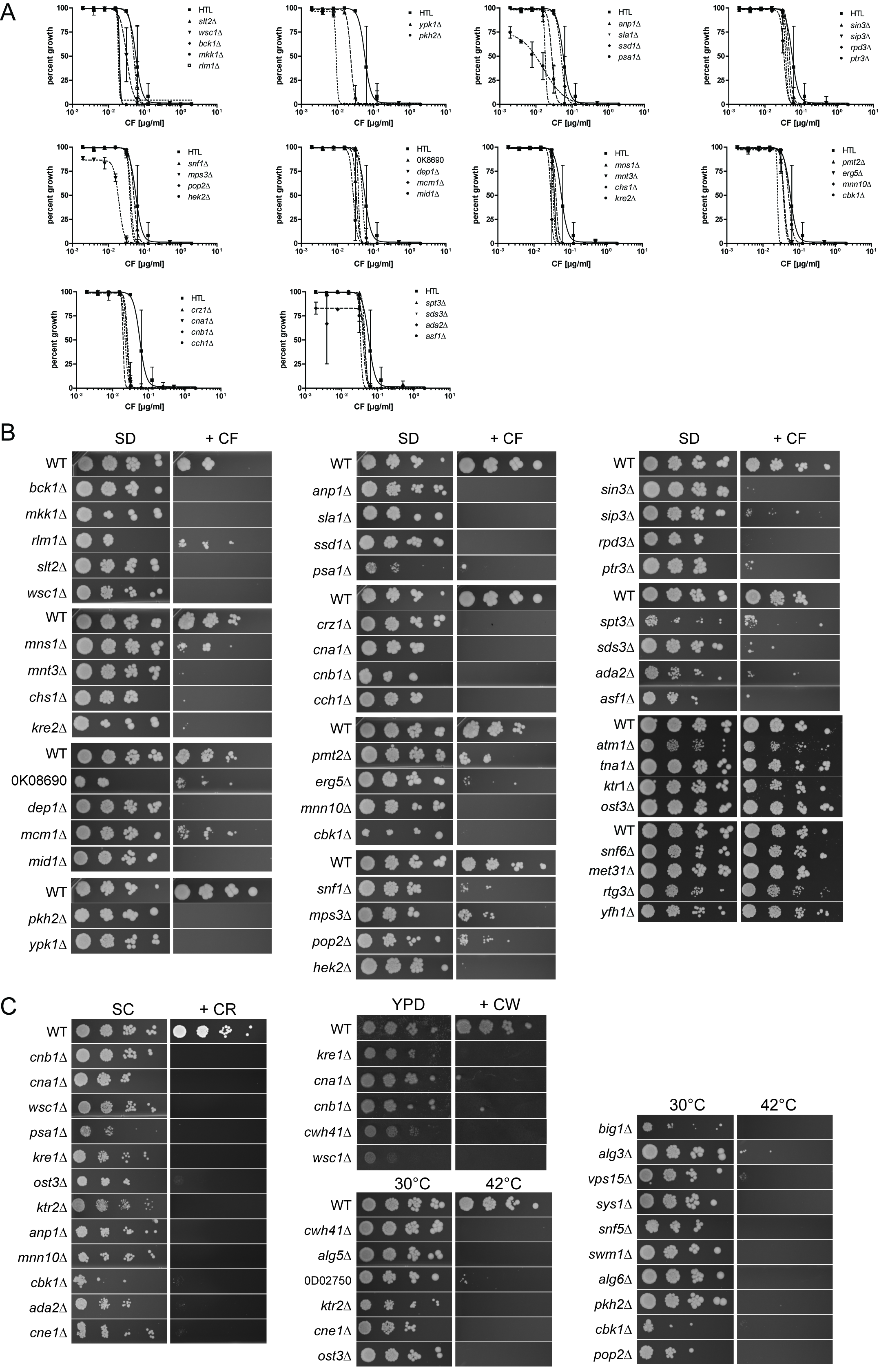

Supplement: Figure S7 — Sensitivity of C. glabrata deletion strains to cell integrity stressors. Deletion strain sensitivities were tested against CF, CW, CR, and 42°C in liquid culture and on plates. (A) Growth susceptibilities of C. glabrata strains to CF. Susceptible strains identified in the primary robotic screenings were subjected to IC50 determination. Overnight cultures were grown to OD600 of 1.0 and a microdilution assay was carried out in YPD to determine IC50 as described in Materials & Methods. The optical density (OD) of each well was determined at 600 nm on a microplate reader after 24 h and 48 h of incubation at 30°C. Each strain was tested in triplicates and mean values normalized to the untreated control were plotted against the antifungal concentration. The IC50 was calculated by nonlinear regression (curve fit), using GraphPad Prism. Shown are data of 48 h measurement. Bars denote standard deviations. (B) Confirmation of CF sensitivity on plates. Strains were spotted in serial dilutions on synthetic medium supplemented with 120 ng/ml caspofungin (CF) and growth was monitored over 3 days at 30°C. (C) Congo Red (CR), heat and Calcofluor White (CW) sensitivity on plates. Strains were spotted in serial dilutions on synthetic medium supplemented with 250 µg/ml Congo Red (CR) or 50 µg/ml CW and growth was monitored over 3 days at 30°C. For heat stress assay cells were grown at 42°C over three days. (TIF) [file ppat.1004211.s007.tif]

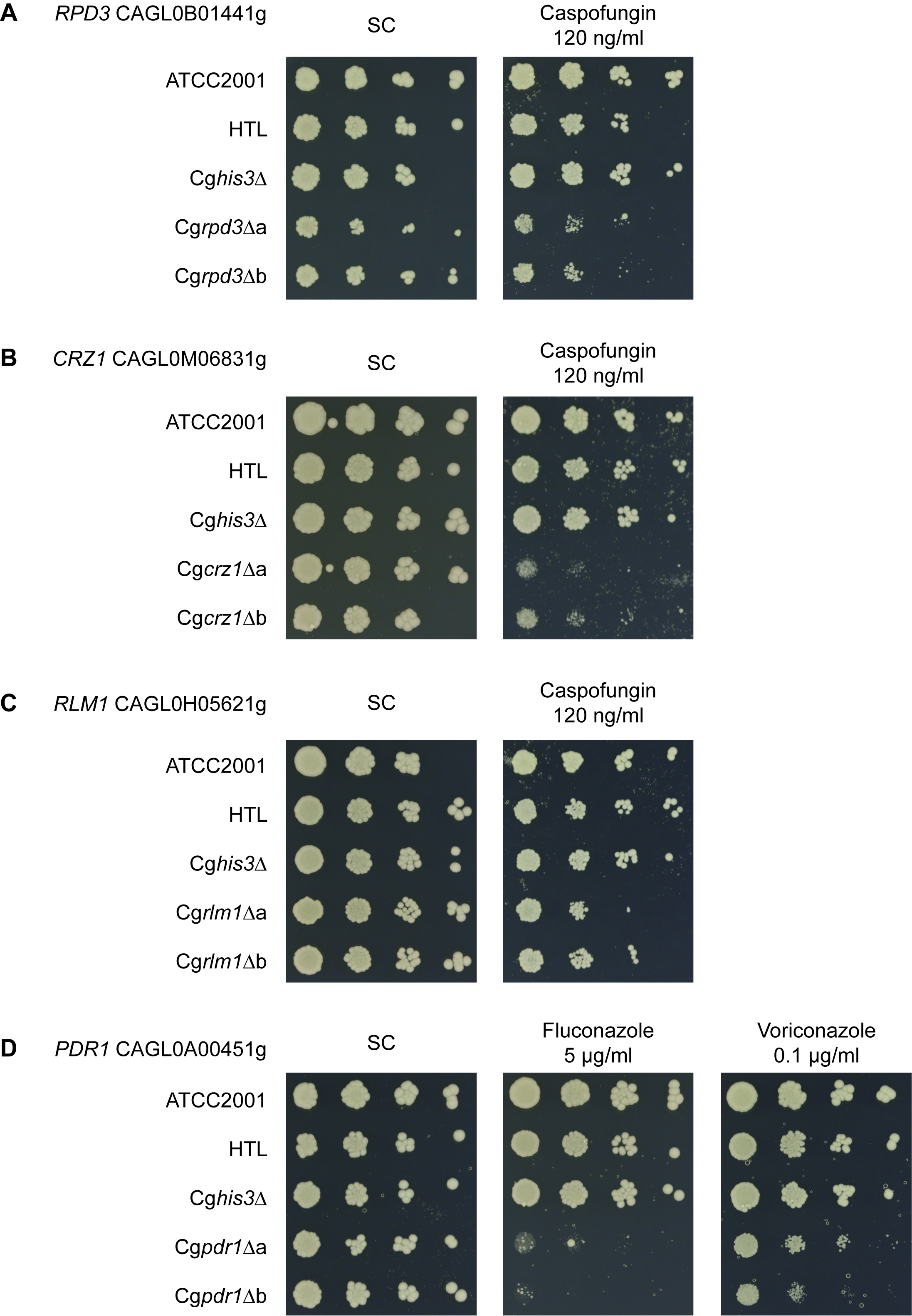

Supplement: Figure S8 — Caspofungin and azole sensitivities of C. glabrata deletions in different genetic backgrounds. Sensitivities of deletion strains constructed in the HTL (a) and the his3 (b) background strains were tested for caspofungin (CF), fluconazole (Flc) and voriconazole (Vor) susceptibility on plates. Strains were spotted in serial dilutions on synthetic medium supplemented with the indicated drug concentrations, and growth monitored for 3 days at 30°C. Screening was performed for (A) RPD3, (B) CRZ1, (C) RLM1 and (D) PDR1. (TIF) [file ppat.1004211.s008.tif]

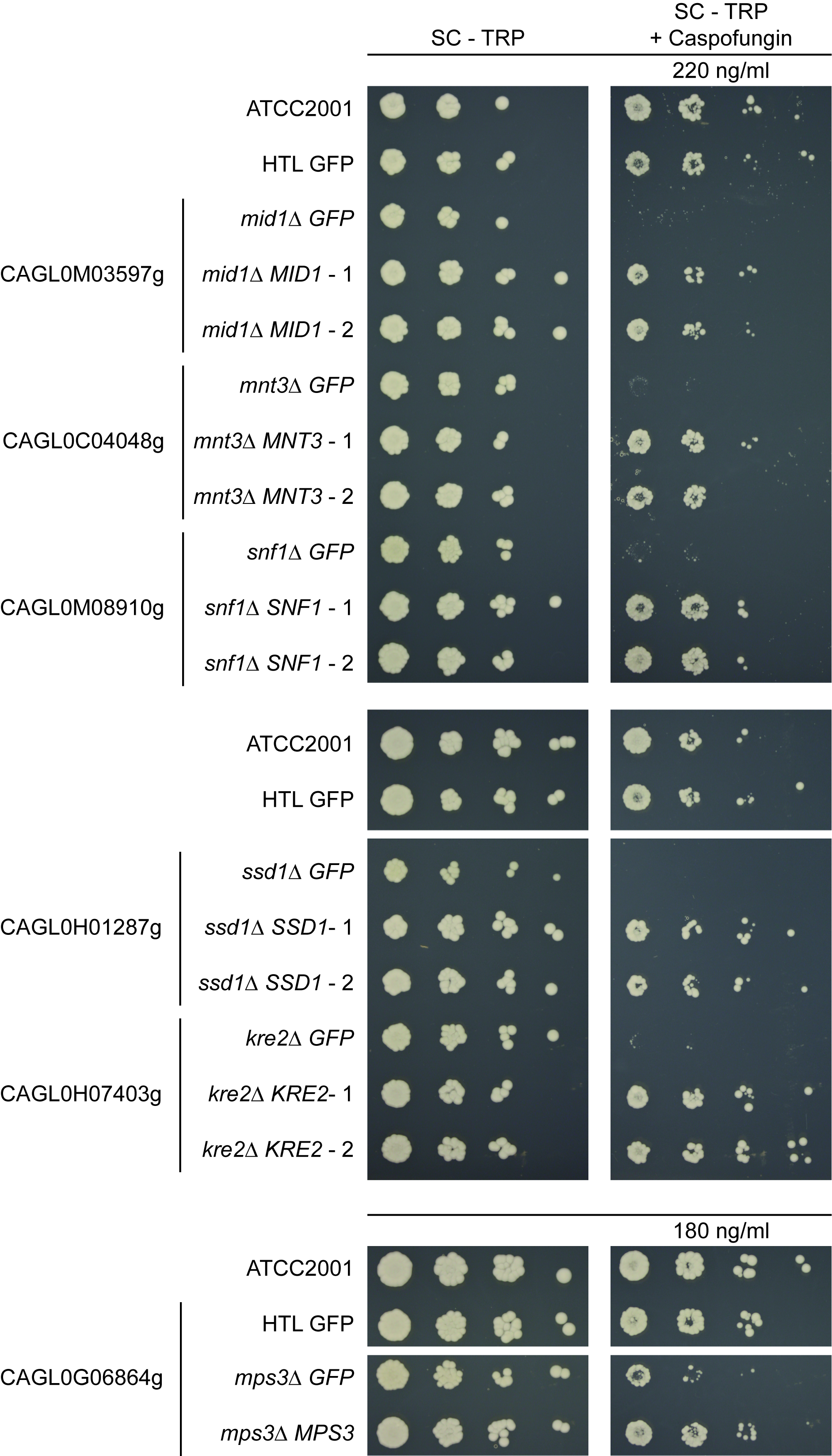

Supplement: Figure S9 — Caspofungin sensitivity of C. glabrata revertant strains. Sensitivities of deletion strains carrying the corresponding wild type gene or a control plasmid containing GFP were tested for caspofungin (CF) susceptibilities on plates. Strains were spotted in serial dilutions on synthetic medium supplemented with CF and growth was monitored over 3 days at 30°C. HTL GFP: HTL control strain carrying GFP-containing plasmid; (TIF) [file ppat.1004211.s009.tif]
