# Supplementary material for: Systematic Phenotyping of a Large-Scale Candida glabrata Deletion Collection Reveals Novel Antifungal Tolerance Genes
Source: PLoS Pathog. 2014 Jun 19;10(6):e1004211. doi: 10.1371/journal.ppat.1004211 (PMC4063973; doi:10.1371/journal.ppat.1004211)
Supplement: Table S1 — Classification of functional gene categories. Genes were selected by their homology to S. cerevisiae based on SGD gene ontology annotations (http://www.yeastgenome.org). C. glabrata orthologues were identified using a BLAST approach. The three best-aligned hits for each gene were saved according to decreasing P values. The orthologues have been remapped using a tree-based approach applying the algorithm described in [50], [53]. A total number of 1047 C. glabrata genes were subjected to gene disruption. (DOC) [file ppat.1004211.s010.doc]

**Table S1. Classification of functional gene categories.** Genes were selected by their homology to *S. cerevisiae* based on SGD gene ontology annotations (www.yeastgenome.org). *C. glabrata* orthologues were identified using a BLAST approach. The three best-aligned hits for each gene were saved according to decreasing P values. The orthologues have been remapped using a tree-based approach applying the algorithm described in [50, 53]. A total number of 1047 *C. glabrata* genes were subjected to gene disruption.

| **Functional category** | **Genes and pathways** | **Number** |
| --- | --- | --- |
| Signal transduction | MAP kinase signaling, HOG, TOR, RIM, PKA, PKC, heat | 100 |
| Transcriptional regulation | Putative transcription factors | 197 |
| Membrane transporters | PDR network, ABC transporters, permeases | 81 |
| Cell wall organization | Synthesis of glucan, mannan, chitin; glycosylation, adhesins, GPI-anchored proteins | 193 |
| Membrane proteins | Transmembrane, integral, lipid homeostasis, ergosterol pathway | 45 |
| Chromatin function | Chromatin / histone modifiers, telomeric silencing | 51 |
| Vacuole, Golgi | Vesicle-mediated transport, vacuole organization | 61 |
| Other genes | Iron and heavy metal metabolism, peroxisome biogenesis, oxidative catabolism, no orthologue in *S. cerevisiae* | 319 |
