# Supplementary material for: Systematic Phenotyping of a Large-Scale Candida glabrata Deletion Collection Reveals Novel Antifungal Tolerance Genes
Source: PLoS Pathog. 2014 Jun 19;10(6):e1004211. doi: 10.1371/journal.ppat.1004211 (PMC4063973; doi:10.1371/journal.ppat.1004211)
Supplement: Table S2 — C. glabrata background recipient strains used in this study. The triple auxotrophic strain HTL and isogenic single and double deletion strains were generated in the sequenced reference strain ATCC2001. Strain HTL was used as recipient strain for the generation of the deletion mutants in the strain library. All other strains of the deletion strain collection are deposited in the C. glabrata deletion strain library (http://funpath.cdl.univie.ac.at) and listed in Table S3. (DOC) [file ppat.1004211.s011.doc]

**Table S2. *C. glabrata* background recipient strains used in this study.** The triple auxotrophic strain HTL and isogenic single and double deletion strains were generated in the sequenced reference strain ATCC2001. Strain HTL was used as recipient strain for the generation of the deletion mutants in the strain library. All other strains of the deletion strain collection are deposited in the *C. glabrata* deletion strain library ([http://funpath.cdl.univie.ac.at](http://funpath.cdl.univie.ac.at/)) and listed in Supplemental Table S3.

| **Strain** | **Genotype** | **Reference** |
| --- | --- | --- |
| ATCC 2001 | *C. glabrata* wild type strain | www.attc.org |
| HTL | *his3*∆::FRT *leu2*∆::FRT *trp1*∆::FRT | This study |
| H | Isogenic to ATCC2001; *his3*∆::FRT | This study |
| T | Isogenic to ATCC2001; *trp1*∆::FRT | This study |
| L | Isogenic to ATCC2001; *leu2*∆::FRT | This study |
| HT | Isogenic to ATCC2001; *his3*∆::FRT *trp1*∆::FRT | This study |
| TL | Isogenic to ATCC2001; *trp1*∆::FRT *leu2*∆::FRT | This study |
| HL | Isogenic to ATCC2001; *his3*∆::FRT *leu2*∆::FRT | This study |
| HTL reference | *his3*∆::FRT *leu2*∆::FRT *trp1*∆::*NAT1* (barcode) | This study |

KK004 clinical isolate

KK006 clinical isolate

KK045 clinical isolate

KK047 clinical isolate
