# Supplementary material for: Systematic Phenotyping of a Large-Scale Candida glabrata Deletion Collection Reveals Novel Antifungal Tolerance Genes
Source: PLoS Pathog. 2014 Jun 19;10(6):e1004211. doi: 10.1371/journal.ppat.1004211 (PMC4063973; doi:10.1371/journal.ppat.1004211)
Supplement: Table S7 — Fitness distribution of C. glabrata knock-out mutants. The Table shows the distribution of fitness phenotypes. Strains with a standard deviation (SD) above 0.1 or an absolute difference in fitness of more than 0.1 between two biological replicates were excluded from detailed analysis. Classification was based on the number of standard deviations between the fitness of a single strain and the average fitness. A total of 504 deletion strains were scored. (DOC) [file ppat.1004211.s016.doc]

**Table S7. Fitness distribution of *C. glabrata* knock-out mutants.** The Table shows the distribution of fitness phenotypes. Strains with a standard deviation (SD) above 0.1 or an absolute difference in fitness of more than 0.1 between two biological replicates were excluded from detailed analysis. Classification was based on the number of standard deviations between the fitness of a single strain and the average fitness. A total of 503 deletion strains were scored.

| **Relative fitness** | **No of SD above/below average relative fitness** | **Number of genes (%)** |
| --- | --- | --- |
| F < 0.887 | -3 or less | 50 (9.9%) |
| 0.887 < F < 0.925 | -2 to -3 | 20 (4.0%) |
| 0.925 < F < 0.962 | -1 to -2 | 44 (8.7%) |
| 0.962 < F < 1.038 | -1 to 1 | 275 (54.6%) |
| 1.038 < F < 1.076 | 1 to 2 | 80 (15.9%) |
| 1.076 < F < 1.113 | 2 to 3 | 28 (5.6%) |
| 1.113 < F | 3 or more | 6 (1.2%) |
