# Supplementary material for: Systematic Phenotyping of a Large-Scale Candida glabrata Deletion Collection Reveals Novel Antifungal Tolerance Genes
Source: PLoS Pathog. 2014 Jun 19;10(6):e1004211. doi: 10.1371/journal.ppat.1004211 (PMC4063973; doi:10.1371/journal.ppat.1004211)
Supplement: Table S10 — Biofilm fitness distribution of C. glabrata knock-out mutants. Summary of biofilm fitness phenotypes. Strains with a standard deviation (SD) above 0.3, or an absolute difference in biofilm fitness of more than 0.5 between replicates were excluded. Classification was based on the number of standard deviations between the fitness of a single strain and the average fitness. A total of 420 deletion strains were subjected to biofilm fitness analysis. (DOC) [file ppat.1004211.s019.doc]

**Table S10. Biofilm fitness distribution of *C. glabrata* knock-out mutants.** Summary of the distribution of biofilm fitness phenotypes. Strains with a standard deviation (SD) above 0.3, or an absolute difference in biofilm fitness of more than 0.5 between replicates were excluded. Classification was based on the number of standard deviations between the fitness of a single strain and the average fitness. A total of 420 deletion strains were subjected to biofilm fitness analysis.

| **Relative biofilm fitness** | **No of SD above/below average relative biofilm fitness** | **Number of genes (%)** |
| --- | --- | --- |
| Fb < 0.628 | -3 or less | 22 (5.2%) |
| 0.628 < Fb < 0.748 | -2 to -3 | 22 (5.2%) |
| 0.748 < Fb < 0.868 | -1 to -2 | 62 (14.8%) |
| 0.868 < Fb < 1.108 | -1 to 1 | 204 (48.6%) |
| 1.108 < Fb < 1.227 | 1 to 2 | 75 (17.9%) |
| 1.227 < Fb < 1.347 | 2 to 3 | 15 (3.6%) |
| 1.347 < Fb | 3 or more | 20 (4.8%) |
