# Supplementary material for: Systematic Phenotyping of a Large-Scale Candida glabrata Deletion Collection Reveals Novel Antifungal Tolerance Genes
Source: PLoS Pathog. 2014 Jun 19;10(6):e1004211. doi: 10.1371/journal.ppat.1004211 (PMC4063973; doi:10.1371/journal.ppat.1004211)
Supplement: Table S11 — Plasmids used in this study. (DOC) [file ppat.1004211.s020.doc]

**Table S11. Plasmids used in this study.**

| **Strain** | ***Cg* ORF** | **Reference** |
| --- | --- | --- |
| pDEST-CgACT-pTDH3-GFP |  | This study |
| pDEST-CgACT-pTDH3-SNF1 | CAGL0M08910g | This study |
| pDEST-CgACT-pTDH3-MID1 | CAGL0M03597g | This study |
| pDEST-CgACT-pTDH3-SSD1 | CAGL0H01287g | This study |
| pDEST-CgACT-pTDH3-KRE2 | CAGL0H07403g | This study |
| pDEST-CgACT-pTDH3-MPS3 | CAGL0G06864g | This study |
| pDEST-CgACT-pTDH3-MNT3 | CAGL0C04048g | This study |
|  |  |  |
|  |  |  |
|  |  |  |
|  |  |  |
|  |  |  |
|  |  |  |
|  |  |  |
|  |  |  |
|  |  |  |
|  |  |  |
