# Supplementary material for: Systematic Phenotyping of a Large-Scale Candida glabrata Deletion Collection Reveals Novel Antifungal Tolerance Genes
Source: PLoS Pathog. 2014 Jun 19;10(6):e1004211. doi: 10.1371/journal.ppat.1004211 (PMC4063973; doi:10.1371/journal.ppat.1004211)
Supplement: Table S12 — Revertant strains used in this study. (DOC) [file ppat.1004211.s021.doc]

**Table S12. Revertant strains used in this study.**

| **Strain** | **Genotype** | **Reference** | |  |
| --- | --- | --- | --- | --- |
| CEG37 | *his3*∆::FRT *leu2*∆::FRT *trp1*∆::FRT / pCgACT-*P_TDH3_-GFP* | | This study | |
| CEG55 | *his3*∆::FRT *leu2*∆::FRT *trp1*∆::FRT *mid1*∆::NAT1 / pCgACT-*P_TDH3_-GFP* | | This study | |
| CEG71 | *his3*∆::FRT *leu2*∆::FRT *trp1*∆::FRT *ssd1*∆::NAT1 / pCgACT-*P_TDH3_-GFP* | | This study | |
| CEG83 | *his3*∆::FRT *leu2*∆::FRT *trp1*∆::FRT *snf1*∆::NAT1 / pCgACT-*P_TDH3_-GFP* | | This study | |
| CEG77 | *his3*∆::FRT *leu2*∆::FRT *trp1*∆::FRT *kre2*∆::NAT1 / pCgACT-*P_TDH3_-GFP* | | This study | |
| CEG97 | *his3*∆::FRT *leu2*∆::FRT *trp1*∆::FRT *mps3*∆::NAT1 / pCgACT-*P_TDH3_-GFP* | | This study | |
| CEG93 | *his3*∆::FRT *leu2*∆::FRT *trp1*∆::FRT *mnt3*∆::NAT1 / pCgACT-*P_TDH3_-GFP* | | This study | |
| CEG57 | *his3*∆::FRT *leu2*∆::FRT *trp1*∆::FRT *mid1*∆::NAT1 / pCgACT-*P_TDH3_-MID1* | | This study | |
| CEG73 | *his3*∆::FRT *leu2*∆::FRT *trp1*∆::FRT *ssd1*∆::NAT1 / pCgACT-*P_TDH3_-SSD1* | | This study | |
| CEG85 | *his3*∆::FRT *leu2*∆::FRT *trp1*∆::FRT *snf1*∆::NAT1 / pCgACT-*P_TDH3_-SNF1* | | This study | |
| CEG61 | *his3*∆::FRT *leu2*∆::FRT *trp1*∆::FRT *kre2*∆::NAT1 / pCgACT-*P_TDH3_-KRE2* | | This study | |
| CEG99 | *his3*∆::FRT *leu2*∆::FRT *trp1*∆::FRT *mps3*∆::NAT1 / pCgACT-*P_TDH3_-MPS3* | | This study | |
| CEG95 | *his3*∆::FRT *leu2*∆::FRT *trp1*∆::FRT *mnt3*∆::NAT1 / pCgACT-*P_TDH3_-MNT3* | | This study | |
|  |  | |  | |
|  |  | |  | |
|  |  | |  | |
|  |  | |  | |
