# Supplementary material for: Systematic Phenotyping of a Large-Scale Candida glabrata Deletion Collection Reveals Novel Antifungal Tolerance Genes
Source: PLoS Pathog. 2014 Jun 19;10(6):e1004211. doi: 10.1371/journal.ppat.1004211 (PMC4063973; doi:10.1371/journal.ppat.1004211)
Supplement: Table S13 — Oligonucleotides used for construction of revertant strains in this study. (DOCX) [file ppat.1004211.s022.docx]

**Table S13: Oligonucleotides used for construction of revertant strains in this study**

| GENE | ORF | FWD | REV |
| --- | --- | --- | --- |
| MID1 | CAGL0M03597g | GGGACAAGTTTGTACAAAAAAGCAGGCTTgATGCTGCTGTGGGTATTGTTCTTGTACTTG | GGGACCACTTTGTACAAGAAAGCTGGGTCTATTTGTGTATCTTAAGGTAAGATGCATT |
| SNF1 | CAGL0M08910g | GGGACAAGTTTGTACAAAAAAGCAGGCTTgATGGAGAACAAGGAGCATCATCACCACCAC | GGGACCACTTTGTACAAGAAAGCTGGGTCTAGTTTCCTTGACTGTTTACAGCCAATTC |
| SSD1 | CAGL0H01287g | GGGACAAGTTTGTACAAAAAAGCAGGCTTGATGTCGAAGTTTCATCGCCAGGATGGTGCC | GGGACCACTTTGTACAAGAAAGCTGGGTTTAGTGTTTTGGTTCCACTTTCATGAAGGG |
| MPS3 | CAGL0G06864g | GGGACAAGTTTGTACAAAAAAGCAGGCTTgATGACAGTGGAAGAATCCGGAGTGAAGGAT | GGGACCACTTTGTACAAGAAAGCTGGGTTTAAATGTCTAGTTCATCCTGCCCAAAAGC |
| KRE2 | CAGL0H07403g | GGGACAAGTTTGTACAAAAAAGCAGGCTTgATGGCTATTTTCTTGTCAAAGAGGCTGATC | GGGACCACTTTGTACAAGAAAGCTGGGTTTAATCGCTGTAATCTTCCCAATTGCTTGG |
| MNT3 | CAGL0C04048g | GGGACAAGTTTGTACAAAAAAGCAGGCTTgATGAATCGCAGGACTAAGAGTCTGCTGAGA | GGGACCACTTTGTACAAGAAAGCTGGGTTCAAATTTTTGCTGAGTTCCAAAATTCAGA |
|  |  |  |  |
